# A modular hydrogel system with independent control of bioadhesion, fibrosis, and stiffness

**DOI:** 10.1126/sciadv.aee3894

**Published:** 2026-06-24

**Authors:** Jiawei Yang, Yichao Zhao, William J. Jeang, Steffen Pabel, Bryan M. Wong, Rajith Manan, Matthias Nahrendorf, Robert Langer, Daniel G. Anderson

**Affiliations:** ^1^ David H Koch Institute for Integrative Cancer Research, Massachusetts Institute of Technology, Cambridge, MA 02139, USA.; ^2^ Department of Anesthesiology, Boston Children’s Hospital, Boston, MA 02115, USA.; ^3^ Department of Mechanical and Materials Engineering, Worcester Polytechnic Institute, Worcester, MA 01609, USA.; ^4^ Department of Biomedical Engineering, Worcester Polytechnic Institute, Worcester, MA 01609, USA.; ^5^ Department of Materials Science and Engineering, Massachusetts Institute of Technology, Cambridge, MA 02139, USA.; ^6^ Center for Systems Biology, Massachusetts General Hospital and Harvard Medical School, Boston, MA 02114, USA.; ^7^ Department of Biological Engineering, Massachusetts Institute of Technology, Cambridge, MA 02139, USA.; ^8^ Department of Chemical Engineering, Massachusetts Institute of Technology, Cambridge, MA 02139, USA.; ^9^ Institute of Medical Engineering and Science, Massachusetts Institute of Technology, Cambridge, MA 02139, USA.

## Abstract

Hydrogels that adhere to biological tissues and resist fibrosis are required to provide both optimal functionality and appropriate stiffness on diverse soft tissues to achieve therapeutic efficacy and biocompatibility. However, their performance is often limited by an intrinsic trade-off between functionality and stiffness. Through the incorporation of polymer brush coatings, we develop a modular hydrogel system to enable independent control of functionality and stiffness. By tailoring coating chemistry, coating thickness, and hydrogel network topology, we obtain consistent bioadhesion (~100 joules per square meter) and fibrosis suppression across the full stiffness range of soft tissues (1 kilopascal to 1 megapascal). Using this approach, we design a hydrogel that can maintain stable adhesion in vivo on a beating mouse heart and a hydrogel with no fibrotic capsule in immunocompetent mice over 40 days. This modular system offers a customizable approach for designing functional implants with tailored mechanical properties.

## Teaser

A modular hydrogel system enables consistent bioadhesion and antifibrosis across the full stiffness range of soft tissues.

## INTRODUCTION

Hydrogels have emerged as an important class of biomaterials due to their tissue-like mechanical and chemical properties and their flexibility in tailoring these properties (*1*–*4*). In many therapeutic applications, such as tissue repair (*5*–*7*), bioelectronic therapy (*8*–*10*), regenerative medicine (*11*–*13*), and drug delivery (*14*–*18*), hydrogels are required to adhere to biological tissues to enable localized and targeted therapies and to resist fibrosis to allow continuous and long-term therapies. As a result, bioadhesive hydrogels (*19*, *20*) and antifibrotic hydrogels (*21*, *22*) have been broadly developed to support these therapies.

Hydrogels have been implanted at various sites throughout the body, including subcutaneously, intraperitoneally, on the surface of a beating heart, and inside the brain (*1*, *2*). Implantation sites can have markedly distinct stiffness, spanning three orders of magnitude, from the brain and pancreas (~1 kPa), the heart and liver (~10 kPa), and skeletal muscle and cartilage (~100 kPa to ~1 MPa) (*23*, *24*). Low stiffness bioadhesive hydrogels (1 to 10 kPa) conform to soft or even dynamic tissues without imposing mechanical constraint, enabling tissue sealing, drug delivery, and bioelectronic recording and stimulation (*6*, *8*, *18*, *25*). However, in applications requiring mechanical stimulation, such as muscle regeneration, the low stiffness cannot effectively transmit force to the underlying tissue (*11*, *13*, *26*). Although increasing stiffness can enhance force transmission, it often compromises adhesion performance (*27*, *28*). Antifibrotic hydrogels with stiffness in the range of 10 to 100 kPa have been developed to enable long-term drug delivery, cell therapy, and biosensing (*1*, *17*, *29*). However, increasing stiffness beyond this range to meet the mechanical demands of load-bearing applications or plastic surgery often induces a stiffness-driven foreign body response (FBR) (*29*, *30*), undermining therapeutic efficacy. These limitations highlight a critical challenge in hydrogel development: the trade-off between functionality and stiffness.

**Fig. 1.**
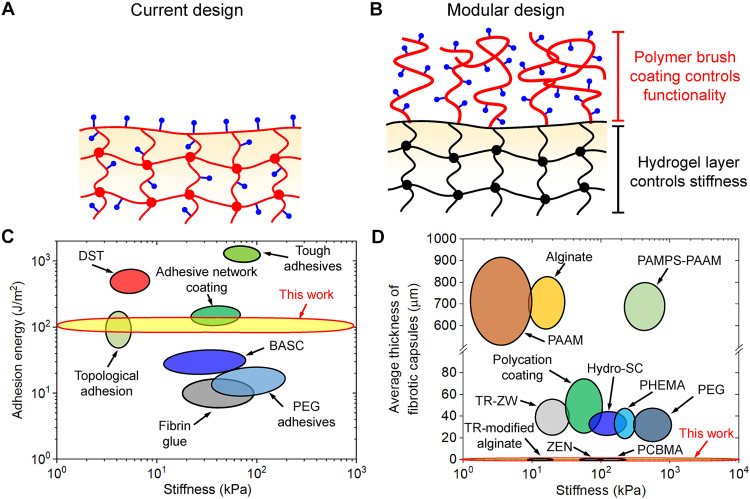
Design and performance of the modular hydrogel system. (**A**) Hydrogel chemical requirements often result in a trade-off between stiffness and functionality. (**B**) The modular hydrogel system decouples the chemistries for functionality and stiffness, by using polymer brush chemistry to control functionality and hydrogel chemistry to control stiffness. The schematic shows the surface-grown brush that contributes to the coating. (**C**) Comparison of various bioadhesive hydrogels (*5*, *6*, *19*, *25*, *45*, *47*–*49*, *73*) and this work in a chart of adhesion energy and stiffness across three orders of magnitude. (**D**) Comparison of various hydrogels and this work in a chart of average fibrotic capsule thickness and stiffness across four orders of magnitude. The data (*21*, *22*, *29*, *37*, *38*, *53*–*57*) include hydrogel implantation in the intraperitoneal and subcutaneous space of mice and rats between 14 and 60 days. Acronyms: DST, bioadhesive double-sided tape; BASC, bioadhesive semiconducting hydrogel; adhesive network coating, polydopamine, poly(acrylic acid-*co*-*N*-hydroxysuccinimide ester); TR, triazole; ZW, zwitterionic; PEG, poly(ethylene glycol); PCBMA, poly(carboxybetaine methacrylate); PAAM, poly(acrylamide); PHEMA, poly(2-hydroxyethyl methacrylate); PAMPS, poly(2-acrylamide-2-metyl-propane sulfonic acid); ZEN, ZW-elastomeric-networked hydrogel; hydro-SC, hydrogel semiconductors; polycation coating, poly(β-amino alcohol), poly-l-lysine, poly-d-lysine, poly-l-ornithine.

A fundamental challenge with hydrogel design is the need for materials that can simultaneously fulfill two distinct goals—establishing a particular network topology for desired stiffness while also incorporating biofunctional groups. For biofunctional design, carboxylic acid groups (*5*, *31*), *N*-hydroxysuccinimide (NHS) ester (*6*, *25*, *32*), and catechol (*33*, *34*) chemistries are used to create strong bonding with tissues, and triazole (TR) (*17*, *22*, *29*, *35*, *36*), zwitterionic (ZW) (*21*, *37*), and TR-ZW (*38*) chemistries have been leveraged to modulate the FBR. For stiffness design, hydrogel chemistries also need to be tailored for different network topologies to broadly vary stiffness. A single-network topology generally yields stiffness below 100 kPa, a double-network (DN) topology increases stiffness to 100 kPa to 1 MPa, and a semicrystalline topology further enhances stiffness to 1 MPa to 10 MPa (*39*). Hydrogel chemistries of weak interchain interactions often form a single-network topology, such as polyacrylamide (PAAM) hydrogels, whereas those of strong interchain interactions can form a semicrystalline topology, such as poly(vinyl alcohol) (PVA) hydrogels (*39*). However, the hydrogel chemistries required for functional design—bioadhesion and antifibrosis—are often coupled with those needed to establish specific network topologies for stiffness control (Fig. 1A). As a result, current bioadhesive and antifibrotic hydrogels can reach optimal functionality within a narrow stiffness range and fail to maintain consistent performance across the full stiffness range of soft tissues.

Here, we develop a modular hydrogel system to decouple the design of functionality and stiffness (Fig. 1B). This system comprises three independently controllable parameters: coating chemistry, which controls the bioadhesive and antifibrotic function; coating thickness, which modulates the extent of functional performance; and hydrogel network topology, which determines stiffness. We use a microscale NHS ester coating for bioadhesion and a nanoscale ZW coating for antifibrosis and apply them on hydrogels with single-network and semicrystalline topologies. Ex vivo porcine tissue adhesion tests, in vivo adhesion tests on a beating mouse heart, and in vivo implantation tests in immunocompetent mice demonstrate that these hydrogels achieve consistent bioadhesive performance (~100 J/m^2^) (Fig. 1C) and fibrosis suppression (no fibrotic capsule) (Fig. 1D) across a stiffness range spanning three and four orders of magnitude, covering the full stiffness range of soft tissues.

## RESULTS

### Synthesis of the modular hydrogel system

Polymer brush engraftment on hydrogels is generally limited by the choice of hydrogel chemistry (*40*). Poorly controlled brush thickness and weak engraftment can hinder functional performance and lead to debonding (*40*). We seek to develop a polymer brush coating that could be applied to diverse hydrogel chemistries with a controlled thickness and a stable engraftment. To achieve this, we design a three-step synthesis route: (i) The hydrogel undergoes chemical or physical modification to add hydroxyl groups on the surface (fig. S1A). This requirement is generally straightforward as hydroxyl groups are pervasive in hydrogels (*1*, *4*) (fig. S2A). Alternatively, hydrogels can be copolymerized with hydroxyl-bearing monomers, such as (hydroxymethyl)acrylamide (HMAAM), or mixed with hydroxyl-bearing polymers, such as hyaluronic acid (HA) (fig. S2B and C). (ii) Hydroxyl groups on the hydrogel surface are then chemically converted to tertiary bromides by reacting with 2-bromoisobutyryl bromide (BriBr) (fig. S1B). Taking the PVA hydrogel as an example, after chemical modification, the atomic concentration of bromine is ~4.41% on both surfaces (fig. S3, A and B), which estimates ~18% of hydroxyl groups are converted to tertiary bromides. Each bromine atom occupies a surface area of about 0.68 nm^2^ (Supplementary Text and fig. S4). The bromine atoms are uniformly distributed and highly localized on the surface and weakly distributed in the bulk (fig. S3C), due to a larger exposure to chemical reactions near the surface than in the bulk. This modification method is robust and is also applicable for other types of hydrogels, and similar bromine distributions are observed (fig. S2). (iii) Last, a polymer brush coating is generated and covalently anchored on the surface of the hydrogel through in situ initiation and controlled growth of polymers by photo atom transfer radical polymerization (photo-ATRP) (fig. S1C). The tertiary bromide–modified hydrogels are placed in an ATRP solution containing monomers, Cu(II)/ligand complex, and sacrificial initiators. Polymerization initiates at tertiary bromide sites and propagates to grow a surface-anchored polymer chain. The process of ATRP is monitored and characterized by the proton nuclear magnetic resonance (^1^H NMR) (fig. S5). The thickness of polymer brush coating is controlled by varying the ratio of monomers to sacrificial initiators (Supplementary Text), and a lower ratio leads to a shorter polymer brush and a thinner coating thickness. For the same brush coating, the coating thickness is determined by both the surface density of tertiary bromide initiators and the brush length.

**Fig. 2.**
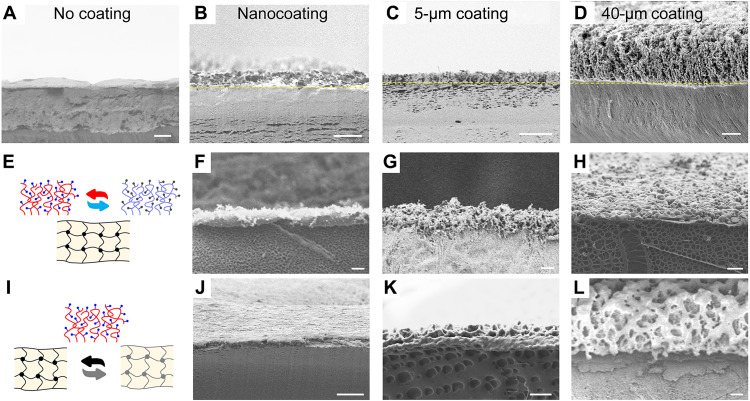
Multiscale polymer brush coating on diverse hydrogels. SEM cross-sectional images of polymer brush coatings across multiple length scales: (**A**) no coating, (**B**) nanocoating, (**C**) 5-μm coating, and (**D**) 40-μm coating. The yellow dashed line delineates the brush-hydrogel boundary. Scale bars, 20 μm. (**E**) Hydrogels are coated with various polymer brushes. SEM cross-sectional images of a PAAM hydrogel with microcoatings of (**F**) PHEMA, (**G**) PAA, and (**H**) PPA. (**I**) The same polymer brush is coated on various hydrogels. SEM cross-sectional images of (**J**) alginate, (**K**) PAAM-PAMPS, and (**L**) PVA hydrogels, with the PMPC microcoating. Scale bars, 20 μm.

To illustrate the multiscale coating capability, we use a poly(acrylic acid) (PAA) brush as a model and coat it on the PVA hydrogel with a thickness ranging from nanometers to micrometers. PVA is a well-controlled polymer that bears the hydroxyl group in each repeated unit, and thereby no physical or chemical step of adding hydroxyl groups is needed. We use scanning electron microscopy (SEM) to evaluate the coating thickness. Without coating, the hydrogel surface exhibits a fabrication roughness in the micrometer scale (Fig. 2A). By adjusting the monomer-to-initiator ratio to 10, the hydrogel surface adds a coarse topography to the original surface (Fig. 2B). The coating thickness is ~5 nm (nanocoating) (Supplementary Text). By adjusting the monomer-to-initiator ratio to 1000, the coating thickness increases to ~5 μm (Fig. 2C). When no sacrificial initiator is added, the coating thickness further increases to ~40 μm (Fig. 2D). Both microscale coatings (microcoating) are sufficiently thick to cover the native surface topography of hydrogels. The distribution of the polymer brush is consistent with that of bromine atoms (fig. S6). Here, we point out that the brush also grows on the hydrogel network near the surface due to the existence of bromine initiators (fig. S3C), but it is buried inside the hydrogel and is not visible in the SEM images. The visible boundary in the SEM images is the boundary between the surface-grown brush layer and the hydrogel substrate. Using different hydrogel substrates can alter the surface density of tertiary bromide initiators and subsequently influence the coating thickness.

We next illustrate that various types of coating can be applied to hydrogels independent of their chemistries (Fig. 2E). We use the PAAM hydrogel to represent a common type of hydrogel as it carries no functional groups for coating. We thus coat the PAAM hydrogel with hydrophilic poly(hydroxyethyl methacrylate) (PHEMA), polyelectrolyte PAA, and hydrophobic poly(propargyl acrylate) (PPA) brushes, and all show a distinct coating-hydrogel two-layer structure (Fig. 2, F to H). Conversely, a given polymer brush coating can be applied on diverse hydrogels (Fig. 2I). We validate this by coating a poly(2-(methacryloyloxy)ethyl 2-(trimethylammonio)ethyl phosphate) (PMPC) brush on hydrogels representing various network topologies, such as alginate of a single-network topology, poly(acrylamide)-poly(2-acrylamido-2-methyl-1-propanesulfonic acid) (PAAM-PAMPS) of a DN topology, and PVA of a semicrystalline topology (Fig. 2, J to L). The coating thickness is controlled to exceed 1 μm to enable clear visualization of the coating-hydrogel structure using SEM. The coating stability is further evaluated by sonicated agitation and wear tests, which show that the coating retains its structural integrity and robust linkage to the hydrogel substrate (fig. S7). Note that using different hydrogel substrates does not affect brush growth as it is controlled by a specific ATRP condition.

### Characterization of the modular hydrogel system

**Fig. 3.**
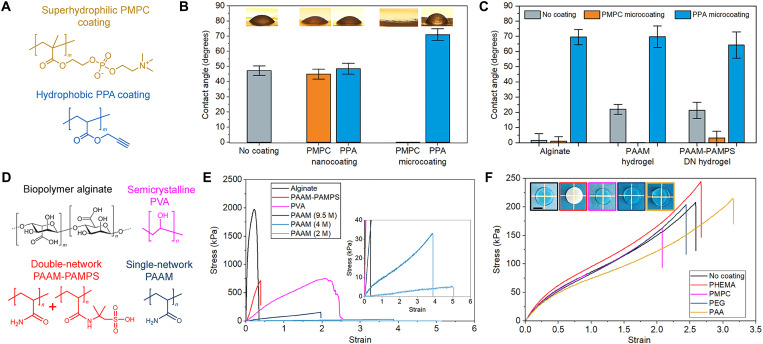
Surface and mechanical properties of the modular hydrogel system. (**A**) Hydrophilic PMPC and hydrophobic PPA coatings are used to illustrate the control of surface properties. Water contact angles of (**B**) PVA hydrogels with the PMPC and PPA nano- and microcoatings (the inset shows a water drop on each coating) and (**C**) other types of hydrogels with the PMPC and PPA microcoatings. (**D**) Hydrogels with four network topologies are used to broadly tune the stiffness. (**E**) Stress-strain curves of hydrogels with the PMPC microcoating. PAAM (2, 4, and 9.5 M) represents the PAAM hydrogels synthesized with 2, 4, and 9.5 M AAM monomers. The inset shows the zoomed region for PAAM (4 and 2 M) hydrogels. (**F**) Stress-strain curves of PAAM hydrogels with various microcoatings. The inset shows the optical images of the respective hydrogels. Scale bar, 4 mm. Error bars denote SDs with five repeated tests.

We first investigate how coating chemistry and thickness influence surface physical properties. We thus select a hydrophilic PMPC brush and a hydrophobic PPA brush that exhibit a large contrast in water affinity to better illustrate the effect (Fig. 3A). We coat a model PVA hydrogel with the two brushes of different thickness and compare the water contact angles. The uncoated PVA hydrogel exhibits a water contact angle of 47°. With a nanocoating (thickness ~ 5 nm), both contact angles remain similar, showing slightly more hydrophilicity by the PMPC coating (contact angle = 45°) and hydrophobicity by the PPA coating (contact angle = 48°). By contrast, with a microcoating (thickness > 1 μm), the difference becomes pronounced—a water drop completely wets the PMPC coating (contact angle = 0°) while beads up on the PPA coating (contact angle = 72°) (Fig. 3B). Because the microcoating is sufficiently thick, the water contact angle should be unaffected by the hydrogel substrates. To confirm this, we apply microcoatings to three other hydrogels, such as alginate, PAAM, and PAAM-PAMPS DN hydrogels. We observe similar trends: The PMPC microcoating reduces the contact angle below 5° for all hydrogel surfaces, whereas the PPA microcoating increases the contact angle to ~70° (Fig. 3C and fig. S8). These observations ascertain that both coating chemistry and thickness control the surface physical properties.

We next assess the stiffness tunability by varying hydrogel network topologies while retaining the same coating. We choose representative network topologies that can cover the most types of hydrogels, such as the alginate and PAAM hydrogels of a single-network topology, the PAAM-PAMPS hydrogel of a DN topology, and the PVA hydrogel of a semicrystalline topology (Fig. 3D). We apply the PMPC microcoating to them to retain the same surface physical properties. The stress-strain curves of these hydrogels reveal distinct mechanical behaviors (Fig. 3E), with stiffness spanning four orders of magnitude, from 1 kPa to 10 MPa (fig. S9). Specifically, the stiffness of single-network PAAM hydrogels varies from ~1 to 100 kPa; the stiffness of semicrystalline PVA hydrogels is about 1 MPa; and the stiffness of PAAM-PAMPS DN hydrogels can reach 2 MPa. Alginate hydrogels show the highest stiffness of ~10 MPa, possibly because the coating process induces substantial hydrogen bonding to stiffen the network (*41*). In addition, other mechanical properties, including stretchability, strength, and work of fracture, also vary by orders of magnitude (fig. S9), demonstrating the capability of broad change of mechanical properties.

We further examine whether coating chemistry affects stiffness. We select the PHEAMA, poly(poly(ethylene glycol) methyl ether acrylate) (PEG-acrylate), PMPC, and PAA microcoatings representing various coating chemistries and coat them on the PAAM hydrogels. The PAAM hydrogel is optically transparent and thus can indicate if the coating can change the optical property. We observe that, except for the PHEMA coating, which appears opaque, the other coatings remain transparent (Fig. 3F, inset). The stress-strain curves of these hydrogels exhibit similar behavior in the small-strain region but with certain deviations in the large-strain region (Fig. 3F). Notably, the stiffness remains ~125 kPa, independent of coating type, whereas other mechanical properties show small variations (fig. S10).

### Design bioadhesive hydrogels with stiffness across the full range of soft tissues

To embed the bioadhesive function into a polymer brush, we graft the brush with the NHS ester groups, which can covalently bond with amines on the tissue to facilitate bioadhesion (*6*, *25*). To make an NHS-grafted brush coating, we first coat a PAA brush as a precursor on a hydrogel and then react the carboxylic acid groups on the PAA brush with 1-ethyl-3-(3-dimethylaminopropyl)carbodiimide (EDC) hydrochloride and *N*-hydroxysulfosuccinimide sodium salt (sulfo-NHS) to graft the NHS ester groups. We use the PAAM and PVA hydrogels that are capable of varying the stiffness from 1 kPa to 1 MPa as substrates and apply the coating to them (fig. S11). In vitro biocompatibility tests using human embryonic kidney (HEK) 293T cells confirm full viability after 24 hours of culture (fig. S12).

The bioadhesive hydrogel is prepared as a dry film, directly placed between two wet tissues (fig. S13A). Upon contact, the dry film rapidly absorbs water on the tissue, softens, and conforms to the tissue (fig. S13B). During this process, the polymer brush may superficially penetrate the tissue network (*5*, *42*) while simultaneously forming hydrogen bonds and peptide bonds with amines on the tissue, resulting in strong and stable adhesion (fig. S13C). We also notice that the surface of the coating is microscopically rough (Fig. 2D and fig. S6), which can cause gaps with the tissue upon contact. Yet the capillarity can overcome the roughness against the stiffness of either the coating or the tissue when the dimensionless number *S*/*E*ε^2^*H* is large, where *S* = γ_1_ + γ_2_ − γ_12_ > 0, the spreading coefficient (γ_1_ and γ_2_ are the surface tensions of the coating and the tissue, and γ_12_ is their interfacial surface tension), *E* is the elastic modulus, ε is the strain, and *H* is the amplitude of the asperities (*27*, *43*). Given *S* ~ 10^−1^ J/m^2^, *E* ~ 10^3^ to 10^6^ Pa, ε ~ 10^−1^, and *H* ~ 10^−6^ m, we estimate that *S*/*E*ε^2^*H* ~ 10 to 10^4^, indicating that capillarity can close the gaps of asperities and enable conformal contact.

**Fig. 4.**
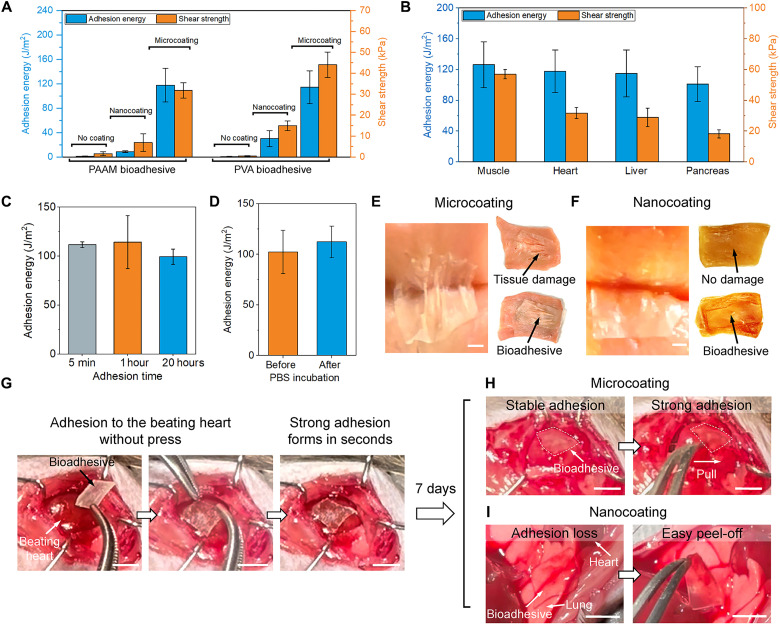
A bioadhesive microcoating enables strong and stable adhesion ex vivo and in vivo. (**A**) Adhesion performance of the PAAM and PVA bioadhesives with nano- and microcoating. (**B**) Adhesion energy and shear strength between various tissues by the PAAM bioadhesive with microcoating. The adhesion is (**C**) stable over time and (**D**) unaffected by the PBS incubation. During the separation of adhered tissues, (**E**) the microcoating causes extensive deformation that leads to tissue damage, whereas (**F**) the nanocoating exhibits a smooth debonding with no tissue damage. (**G**) Surgical procedure of adhering a bioadhesive to a beating mouse heart. In a 7-day implantation, (**H**) the microcoating maintains strong and stable adhesion on the heart, whereas (**I**) the nanocoating loses adhesion and migrates to the lung. Scale bars, 1 mm [(E) and (F)]. Scale bars, 5 mm [(G) to (I)]. Error bars denote SDs with five repeated tests.

To apply the coating on the PAAM hydrogel, the PAAM hydrogel is synthesized by copolymerizing the HMAAM monomers and the AAM monomers to introduce hydroxyl groups that further undergo bromine modification to yield initiators for growing the brush. We vary the AAM-to-HMAAM molar ratios in the synthesis and find that the ratio of 100 gives the optimal adhesion performance (fig. S14, A and B). We subsequently evaluate adhesion by measuring adhesion energy and shear strength (Fig. 4A). We use the porcine heart as a model tissue. The microcoating establishes a tough (adhesion energy > 100 J/m^2^) and strong (shear strength > 35 kPa) adhesion. The SEM image of the interface shows the seamless merging of the bioadhesive and the tissue (fig. S13D). By contrast, the nanocoating attains a weak adhesion (the adhesion energy is 9 J/m^2^, and the shear strength is 11 kPa), and an uncoated PAAM hydrogel exhibits negligible adhesion. This observation can be interpreted by the Lake-Thomas theory of polymer fracture (*44*): Given the similar brush density of nano- and microcoatings, the bonding density is similar, but a thicker coating consists of longer polymers, and breaking them releases a larger elastic energy, thus amplifying the adhesion energy. This adhesion is independent of the hydrogel stiffness of 1 to 100 kPa (fig. S14C). To extend the hydrogel stiffness up to 1 MPa, we use the PVA hydrogel and apply the same microcoating. We attain similar tough and strong adhesion (Fig. 4A). These results evidence that this class of bioadhesive hydrogels achieves consistent adhesion across three orders of magnitude in stiffness (Fig. 1C). By contrast, commercial tissue adhesives such as fibrin glues (e.g., Tisseel) and PEG adhesives (e.g., Coseal and DuraSeal) (*6*, *19*, *45*, *46*), and emerging bioadhesives such as topological adhesion (*47*), bioadhesive double-sided tape (DST) (*6*), bioadhesive semiconducting hydrogel (BASC) (*25*), adhesive network coating (*48*, *49*), and tough adhesives (*5*) illustrate narrow stiffness ranges within one order of magnitude.

The PAAM bioadhesives can bond a range of tissues, including muscle, liver, and pancreas, with consistent adhesion energy (>100 J/m^2^) and shear strength (60 kPa for muscles and 20 to 30 kPa for the liver, heart, and pancreas) (Fig. 4B). The adhesion is stable over time (Fig. 4C) and after phosphate-buffered saline (PBS) incubation (Fig. 4D) and unaffected by the hydrogel thickness (fig. S14D). Upon separation, the microcoating causes substantial deformation at the interface and subsequently damages both the coating and the tissue (Fig. 4E and fig. S13, E and F). By contrast, the nanocoating exhibits a smooth debond with no damage to the tissue (Fig. 4F). These experiments corroborate that the combined brush chemistry for covalent bonding with tissues and the micrometer brush coating lead to strong bioadhesion.

We further examine the in vivo bioadhesion performance. We apply a PAAM bioadhesive to the surface of a beating mouse heart (heart beats 500 to 700 times per minute). Strong adhesion rapidly forms within the time of operation (<1 min) (Fig. 4G and movie S1). After implantation for 7 days, the PAAM bioadhesive with microcoating maintains adhesion with the heart and resists debonding when pulling it (Fig. 4H and movie S2). By contrast, the PAAM bioadhesive with nanocoating loses adhesion and has migrated to the lung, which can be readily peeled off (Fig. 4I and movie S3). These findings underscore the critical role of microcoating in achieving both strong and stable adhesion in vivo.

### Design antifibrotic hydrogels with stiffness across the full range of soft tissues

ZW polymers can create a highly hydrated surface and have been widely used to repel cell adhesion and fibrosis (*21*, *37*). In particular, PMPC has shown a superior antifibrotic performance compared to other ZW polymers (*50*). Therefore, we coat a PMPC brush on hydrogels to provide the antifibrotic function. We use the C57BL/6 mouse model for inducing a strong FBR and choose the PVA hydrogel as a mechanically robust substrate for implantation. PVA hydrogels are coated with both nanoscale and microscale PMPC brushes, and in vitro biocompatibility tests confirm full cell viability (fig. S12).

**Fig. 5.**
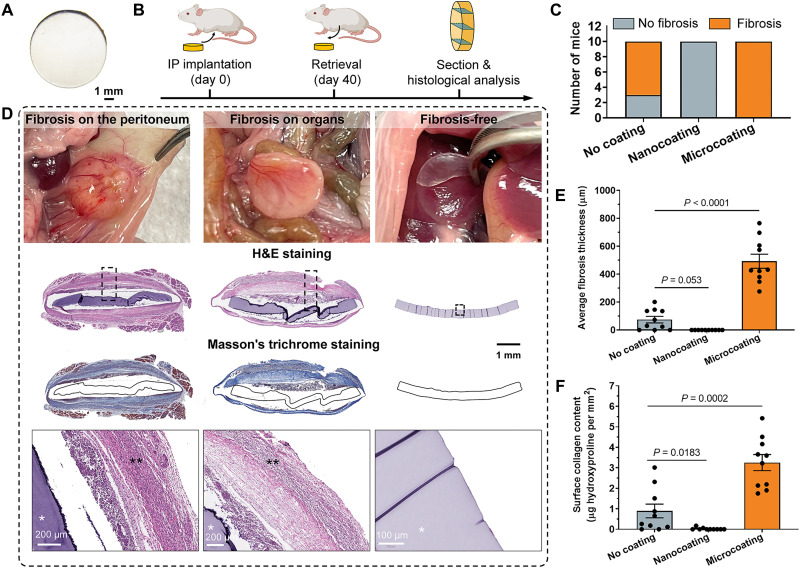
An antifibrotic nanocoating suppresses fibrosis. (**A**) Optical image of a PVA hydrogel with nanocoating. (**B**) PVA hydrogels with no coating, the PMPC nanocoating, and the PMPC microcoating are implanted in the intraperitoneal (IP) space, retrieved after 40 days, sectioned, and performed the histological analysis. (**C**) Summary of fibrosis outcomes. (**D**) Three types of fibrosis are observed: The PVA hydrogels are (i) enveloped by fibrotic capsules on the peritoneum, (ii) on the organs, and (iii) fibrosis-free. The PVA hydrogels are stained with H&E and Masson’s trichrome to characterize fibrosis. Images in the last row are the enlarged view of the areas enclosed by the dashed lines. The single asterisk represents the PVA hydrogel, and the double asterisk represents the fibrotic capsule. Quantitative analysis of fibrosis by the (**E**) average thickness of the fibrotic capsules and (**F**) surface collagen content. Error bars denote means ± SEM. Statistical analysis: two-tailed Student’s *t* tests that compare no coating versus nanocoating and no coating versus microcoating; *P* values are indicated in the figure. Each data point in (E) represents the average thickness at three different sections of a PVA hydrogel. Each data point in (F) represents an individual PVA hydrogel. Total mice = 10 for each group. Created in BioRender. Yang, J. (2026) https://BioRender.com/2twgv9z.

PVA hydrogels are prepared in a disc shape (10 mice for each group) (Fig. 5A), implanted in the intraperitoneal space of mice, retrieved after 40 days, and sectioned for histology analysis (Fig. 5B). Upon retrieval, PVA hydrogels are found at different locations in the peritoneal cavity owing to natural animal movement. By visual inspection, uncoated PVA hydrogels have a mixed fibrosis outcome (7 of 10 are fibrotic encapsulated), PVA hydrogels with nanocoating are all free of fibrotic capsules, whereas PVA hydrogels with microcoating are all encapsulated by thick fibrotic tissues, exhibiting exacerbated fibrosis even compared to uncoated PVA hydrogels (Fig. 5, C and D). Histology assessment [Masson’s trichrome staining and hematoxylin and eosin (H&E) staining] confirms our observation. Fibrosis to PVA hydrogels (7 uncoated and 10 microcoating), either on the peritoneum or on the organs, elicit a severe chronic inflammation, which involves the deposition of immune cells and the formation of a fibrotic capsule comprising extensive collagen and new blood vessels; by contrast, PVA hydrogels with nanocoating recruit a few immune cells with no fibrotic capsules, demonstrating the ability to suppress fibrosis (Fig. 5D). Quantitative analysis shows that the average thickness of fibrotic capsules is about 80 μm for uncoated PVA hydrogels, compared to 0 and 500 μm for PVA hydrogels with nano- and microcoatings (Fig. 5E). Surface collagen content is about 0.87 μg/mm^2^ for uncoated PVA hydrogels, compared to 0.02 and 3.2 μg/mm^2^ for PVA hydrogels with nano- and microcoatings (Fig. 5F).

Given the same brush chemistry and density, and the hydrogel substrates, it seems unexpected that the microcoating with a higher hydration than that of the nanocoating elicits more fibrosis. To understand how the coating thickness influences fibrosis, we further investigate another known parameter for fibrosis, the coating surface roughness (*51*, *52*). Surface roughness is characterized by the root mean square (RMS). Uncoated PVA hydrogels exhibit an RMS of 2 μm, PVA hydrogels with nanocoating have a smoother surface with an RMS of 1.6 μm, but PVA hydrogels with microcoating show a markedly rougher surface with an RMS of 12.5 μm, nearly one order of magnitude rougher (fig. S15). A prior study reported that a surface roughness of ~1 μm minimizes fibrosis in silicone implants (*51*), which suggests that surface smoothness can also contribute to antifibrosis in addition to the ZW chemistry. To substantiate this, we test other hydrogels with the PMPC coating of a similar surface roughness. We synthesize PAAM hydrogels with an RMS of 1.5 μm and alginate hydrogels with an RMS of 1.4 μm and implant them in the same intraperitoneal spacing for 40 days. Upon retrieval, both hydrogels remain largely clean and free of fibrosis (figs. S16 and S17), corroborating that the smooth ZW nanocoating suppresses fibrosis.

These hydrogels span a wide stiffness range across four orders of magnitude, from 1.1 and 134 kPa for PAAM hydrogels, to 1 MPa for PVA hydrogels, and to 22 MPa for alginate hydrogels, and they all suppress fibrosis, evidencing that the antifibrotic performance is independent of hydrogel stiffness (Fig. 1D). By contrast, existing hydrogels, including newly developed antifibrotic hydrogels, either elicit substantial fibrosis (*53*, *54*) or resist fibrosis within narrow stiffness windows (*17*, *21*, *22*, *29*, *37*, *38*, *55*–*57*). Furthermore, we observe that, whereas PVA and PAAM hydrogels remain intact, alginate hydrogels fracture (fig. S17). This is attributed to the low fracture resistance of alginate [fracture toughness of ~1 to 10 J/m^2^ (*58*)] compared to PVA and PAAM hydrogels [fracture toughnesses are 2000 J/m^2^ (*59*) and 500 J/m^2^ (fig. S10E)]. This highlights the importance of high toughness in antifibrotic hydrogel design to ensure implant longevity.

Although our data suggest that the coating roughness plays a bigger role in mitigating fibrosis than coating thickness, hydration state, and stiffness of hydrogels, fibrosis is a complex and dynamic process, and not a single material property can dictate the outcome. Previous studies have shown that a range of material properties, which include stiffness, chemistry, hydration, size, geometry, and roughness, often orchestrate in controlling the degree of fibrosis. For example, a superhydrophilic ZW hydrogel can repel protein adsorption and thus resist fibrosis (*37*), a hydrophobic silicone elastomer can elicit extensive protein adsorption, but it still can mitigate fibrosis by reducing the implant stiffness (*60*) and controlling the roughness around 4 μm (*51*), a TR surface coating that exhibits less hydration and protein resistance than a ZW coating can reduce fibrosis better than the ZW coating (*17*, *50*), and a spherical implant of 1.5 mm in diameter, made across a broad range of materials, including hydrogels, ceramics, metals and plastics, can modulate the fibrosis, independent of their material properties (*61*). These examples corroborate that one material property cannot simply correlate with fibrosis outcomes; in particular, the in vitro protein or cell adsorption tests may also not positively correlate with the in vivo fibrosis tests (*62*).

## DISCUSSION

We present a modular hydrogel system to overcome the intrinsic trade-off between functionality and stiffness commonly encountered in hydrogel development. By restricting the functionality to the polymer brush coating and the stiffness to the hydrogel network topology, the design and control of functionality and stiffness are decoupled. A systematic investigation into polymer brush coating identifies coating thickness as a key parameter for modulating functional performance. The same coating chemistry can lead to opposite outcomes: For bioadhesion, a microcoating confers strong, stable adhesion, whereas a nanocoating results in weak, short-lived adhesion (Fig. 4A); for antifibrosis, a nanocoating resists fibrosis, whereas a microcoating exacerbates fibrosis (Fig. 5, C to F). We apply these coatings to hydrogels of single-network and semicrystalline topologies, achieving consistent functional performance with stiffness across three and four orders of magnitude, spanning the full range of soft tissues (Fig. 1, C and D). This development potentially provides a solution to two major challenges in implantation: bioadhesive-tissue mechanical mismatch (*20*, *63*) and stiffness-driven FBR (*30*, *64*).

Hydrogels in long-term implantation applications need to maintain in vivo functionality, stability, biological and mechanical compatibility, and mechanical durability to withstand the complex and dynamic physiological environment. Most bioadhesives are designed for short-term applications such as wound closure and hemostasis, where short-lived adhesion is sufficient before these applications complete (*5*, *6*, *19*, *20*). However, in long-term implantation, they may suffer premature failure due to unstable adhesion, swelling, and degradation. Our modular system allows for developing bioadhesives based on chemically inert hydrogels to address this issue (Fig. 4). Implant-tissue mechanical compatibility is important to maintain tissue physiological functions, biocompatibility, and therapeutic efficacy (*24*, *63*, *65*). The narrow stiffness range in existing bioadhesive and antifibrotic hydrogels cannot be generally used and suitable for mechanically diverse implantation sites, where tissue stiffness ranges across three orders of magnitude. Our modular system covers the full stiffness range of soft tissues, thus allowing tailoring the hydrogel stiffness to match individual tissue without compromising functionality (Fig. 1, C and D). Our modular system also allows integrating mechanically robust and durable hydrogels to deter wear and fracture. Furthermore, our modular system enables the development of biofunctional hydrogels with tailored mechanical properties beyond stiffness, such as viscoelasticity, viscoplasticity, and fatigue resistance, to achieve mechanical compatibility from the tissue level down to the cellular level (*66*, *67*) and advance therapies that require fine controls of complex mechanical properties.

In summary, this modular design system provides a versatile platform for functional and mechanical control of hydrogel implants, which can be further extended to general biomaterial design. We believe that this platform technology can create new materials for a range of implantation applications.

## MATERIALS AND METHODS

### Materials and reagents

Polymers and monomers for hydrogel synthesis included PVA [weight-average molecular weight (*M*_w_) = 89,000 to 98,000 Da, 99+% hydrolyzed; Sigma-Aldrich, 341584], AAM (Sigma-Aldrich, A9099), HMAAM (TCI America, M0574), AMPS (Sigma-Aldrich, 282731), sodium hyaluronate (*M*_w_ = 500,000 to 749,000 Da; Lifecore, HA700K), and sodium alginate (*M*_w_ = 200,000 to 300,000 Da; PRONOVA SLG100, NovaMatrix). Sodium sulfate (Sigma-Aldrich, 238597) solution was used to strengthen PVA hydrogels. To synthesize P(AAM-*co*-HMAAM), PAAM-HA, and P(AAM-HMAAM)-PAMPS DN hydrogels, *N*,*N*′-methylenebisacrylamide (MBAA; Sigma-Aldrich, M7279) was used as the covalent cross-linker and Irgacure 2959 (Advanced BioMatrix, 5200) was used as ultraviolet (UV) radical initiators. To synthesize alginate hydrogels, calcium chloride (CaCl_2_; Sigma-Aldrich, C4901) and barium chloride (BrCl_2_; Sigma-Aldrich, 217565) were used as ionic cross-linkers.

Chemicals and solvents for the bromine modification of hydrogel surfaces included 4-dimethylaminopyridine (DMAP; TCI America, D1450), BriBr (TCI America, B0607), *N*,*N*′-dimethylformamide (DMF; anhydrous; Sigma-Aldrich, 227056), and acetonitrile (ACN, anhydrous; Sigma-Aldrich, 271004). Monomers for the photoinitiated ATRP included MPC (TCI America, M2005), AA (Sigma-Aldrich, 147230), *N*-acryloxysuccinimide (NHS-acrylate; Acros Organics, AC400300010), PEG-acrylate (Sigma-Aldrich, 454990), HEMA (Sigma-Aldrich, 477028), and PA (TCI America, P2878). All monomer solutions were passed through an alumina column (activated, neutral; Sigma-Aldrich, 799319) to remove inhibitors before use. Ligands for ATRP included copper(II) bromide [Cu(II)Br_2_; TCI America, C2389], copper(II) chloride [Cu(II)Cl_2_; Sigma-Aldrich, 751944], tris[2-(dimethylamino)ethyl]amine (Me_6_TREN; TCI America, T2898), and tris(2-pyridylmethyl)amine (TPMA; TCI America, T2671). Sodium chloride (NaCl; Sigma-Aldrich, S9888) or sodium bromide (NaBr; Sigma-Aldrich, 310506) was added during the ATRP of AA. Ethyl α-bromoisobutyrate (EBIB; Sigma-Aldrich, E14403) and 2-hydroxyethyl 2-bromoisobutyrate (HEBIB; Sigma-Aldrich, 723150) were used as organic-phase and aqueous-phase sacrificial initiators to control the thickness of polymer coating. Fluoresceinamine (fluoresceinamine, isomer I; Sigma-Aldrich, 201626) was used to label NHS-acylate polymer coatings with fluorescence.

Coupling reagents for preparing hydrogel tissue adhesives included EDC hydrochloride (Sigma-Aldrich, E1769) and sulfo-NHS (TCI America, H1304). All porcine tissues for adhesion tests were purchased from a research-grade biological tissue vendor (Sierra Medical).

### Synthesis of hydrogels

#### PVA hydrogels

PVA powders were dissolved in deionized (DI) water (10 wt %) under vigorous stirring and heating at 90°C for 5 hours until the PVA solution became clear. After cooling down to room temperature, the PVA solution was poured into a glass mold with a spacer of various thicknesses, then frozen at −20°C for 1 hour, and thawed at room temperature for 1 hour. This freeze-thaw cycle was repeated three times. Afterward, the PVA hydrogel was soaked in 1 M sodium sulfate solution for 12 hours to enable further hydrogen cross-linking of PVA chains (*68*). Last, the PVA hydrogel was washed thoroughly in DI water to remove sodium sulfate.

#### Alginate hydrogels

A 2 wt % sodium alginate solution was poured into an acrylic mold and immersed in an aqueous solution of 95 mM CaCl_2_ and 5 mM BaCl_2_ to form alginate hydrogels, which were further washed and equilibrated in DI water.

#### PAAM hydrogels (6, 4, and 2 M)

AAM and HMAAM were dissolved in DI water at a molar ratio of 100:1, and then MBAA aqueous solution and Irgacure 2959 methanol solution were added to a final concentration of 0.5 and 1 mM, respectively. The pregel solution was mixed, degassed, poured into a glass mold, and polymerized under UV (UV Lamps, 15 W, 365 nm) for 1 hour. The prepared hydrogels were washed with water to remove unreacted chemicals and further swollen to equilibrium.

#### PAAM hydrogels (9.5 M)

We follow the recipe for making high-strength and high-toughness hydrogels (*69*). Briefly, AAM was dissolved in DI water at a concentration of 9.5 M and mixed with 2 wt % HA. The solution was vigorously stirred and incubated at a temperature of 50°C to yield a clear solution. MBAA aqueous solution and Irgacure 2959 methanol solution were added to a final concentration of 0.1 and 0.045 mM, respectively. The pregel solution was homogeneously mixed, sonicated, degassed, poured into a glass mold, and polymerized under UV for 3 hours. The prepared hydrogels were washed with water to remove unreacted chemicals and further swollen to equilibrium.

#### PAAM-PAMPS DN hydrogels

AMPS was dissolved in DI water at a concentration of 1 M, and then 4 mol % MBAA and 0.1 mol % Irgacure 2959 relative to AMPS were added. The aqueous solution was mixed and poured into an acrylic mold, covered with a glass slide, and exposed to UV for 2 hours. The as-prepared PAMPS network was the first polymer network. Care was taken to transfer it to a pregel solution of the second polymer network, P(AAM-*co*-HMAAM), containing 2 M AAM, 0.02 M HMAAM, 1 mM MBAA, and 0.1 mM Irgacure 2959. The PAMPS network absorbed the pregel solution of the second network and swelled to equilibrium. Subsequently, the swollen PAMPS network was sandwiched between two glass slides and exposed to UV for 2 hours to form the second P(AAM-*co*-HMAAM) network. The first and the second networks were topologically interpenetrated, leading to the P(AAM-*co*-HMAAM)-PAMPS DN hydrogels. The DN hydrogels were washed and immersed in DI water to remove unreacted chemicals and further swollen to equilibrium.

### Surface chemical modification to convert hydroxyl groups to tertiary Br groups

The swollen hydrogels were first fixed between two acrylic frames to constrain their in-plane deformation and only allow thickness deformation, then baked in an oven at a temperature of 50°C overnight, and further dried in a nitrogen flow for another 1 hour. Dry hydrogels were removed from the acrylic frames, placed in a glass vial containing 480 mM DMAP in anhydrous DMF or ACN, flushed with argon gas, cooled down to 0°C in an ice bath, and sealed with a rubber septum. Subsequently, BriBr was added dropwise into the cold reaction solution to a final concentration of 160 mM. The reaction was kept for 12 hours at room temperature. Hydrogels were taken out and thoroughly washed in DI water to remove reaction residues and by-products and then swollen in DI water.

### Polymer brush coating by ATRP

To covalently anchor a polymer brush on the surfaces of hydrogels, we adopted the photo-ATRP technique. UV light of a wavelength of 365 nm can convert Cu(II) to Cu(I) to initiate polymerization. The coating thickness can be controlled by adding the sacrificial HEBIB initiators to the solution. The recipes of ATRP reaction solutions for different polymer coatings are described as follows.

#### PMPC coating

For microcoating, the ATRP solution was composed of 1 M MPC, 1.8 mM Cu(II)Br_2_/TPMA complex (1:6 molar ratio in methanol), and 10 mM NaBr in DI water. For nanocoating, the ATRP solution was composed of 500 mM MPC, 50 mM HEBIB [degree of polymerization (DP) = 10 at 100% conversion], 50 mM Cu(II)Br_2_/TPMA (1:4 molar ratio in methanol), and 10 mM NaBr in DI water.

#### PAA coating

We follow the literature to tune the coating based on both monomer-to-initiator ratio and salt types (*70*). For microcoating, the ATRP solution was composed of 1.46 M AA, 0.1 mM Cu(II)Cl_2_/TPMA (1:4 molar ratio in methanol), and 4 mM NaCl in DI water. The conversion can reach 50% after 18 hours. For nanocoating, the ATRP solution was composed of 1.46 M AA, 14.6 mM HEBIB, 1.46 mM Cu(II)Cl_2_/TPMA (1:4 molar ratio in methanol), and 58.4 mM NaBr in DI water. The conversion was about 10%, corresponding to DP = 10 (fig. S18).

#### PHEMA microcoating

The ATRP solution was composed of 500 mM HEMA and 0.1 mM Cu(II)Br_2_/TPMA complex (1:6 molar ratio in methanol) in DI water.

#### PEG microcoating

The ATRP solution was composed of 500 mM PEG-acrylate and 0.1 mM Cu(II)Br_2_/TPMA complex (1:6 molar ratio in methanol) in DI water.

#### P(NHS-acrylate) microcoating

The ATRP solution was composed of 1 M NHS-acrylate and 0.05 mM Cu(II)Br_2_/Me_6_TREN complex (1:6 molar ratio in DMF) in DMF.

#### PPA microcoating

The ATRP solution was composed of 1 M PA and 0.05 mM Cu(II)Br_2_/Me_6_TREN complex (1:6 molar ratio in DMF) in methanol.

Br-modified hydrogels were immersed in ATRP reaction solutions in a glass vial sealed with a rubber septum, completely deoxygenated by bubbling with argon gas while vigorously stirring for at least 30 min, and exposed to UV light for 20 hours. Afterward, the coated hydrogels were thoroughly washed in abundant water to remove reaction residues.

### XPS characterizations

To prepare x-ray photoelectron spectroscopy (XPS) samples, both pure PVA and Br-modified PVA hydrogels were thoroughly washed and lyophilized to become dry hydrogel samples. The surface chemical composition was characterized by a PHI VersaProbe II x-ray photoelectron spectrometer, operated at a base pressure of <5 × 10^−8^ torr. Three random locations on the hydrogel surfaces were selected and analyzed. The data were processed and quantified using the SmartSoft software with the elemental analysis feature to determine the atomic concentration.

### NMR characterizations

For the aqueous-phase ATRP of MPC, AA, HEMA, and PEG-acrylate, D_2_O was used as the solvent. For the organic-phase ATRP of NHS-acrylate and PA, dimethyl sulfoxide-*d*_6_ (DMSO-*d*_6_) was used as the solvent. We added 1 wt % DMF in all ATRP reaction solutions as an internal standard. We conducted ^1^H NMR for solutions before and after ATRP on a Bruker Avance Neo spectrometer, operating at 500.34 MHz. The polymerization consumed monomers, which showed decreased peaks for protons adjacent to acrylates or methacrylates. The conversion is calculated as the peak area difference of the protons before and after ATRP divided by the peak area before ATRP.

### SEM imaging

All hydrogel samples were lyophilized and sputter coated with 10 nm of gold before imaging. The surface morphologies of hydrogel samples were characterized by a ZEISS Sigma 300 scanning electron microscope (Germany). Surface and cross-sectional views of hydrogel samples were obtained by carefully adjusting the angles for optimal view. Elemental distributions were characterized by SEM coupled with energy-dispersive spectroscopy (EDS) (ZEISS Gemini 450, Germany).

### Polymer coating stability test

PVA hydrogels with PMPC microcoating were placed in an ultrasonic bath for 30 min to test the coating stability and worn against a metal plate in the water using a rheometer (ARES-G2, TA Instrument, spin rate = 1 rad/s, normal pressures = 1 and 10 kPa) to test wear stability. SEM images were subsequently taken to examine the surface and the structure and compare them with the pristine coating.

### Fluorescence labeling of polymer coatings

PVA hydrogels with P(NHS-acrylate) microcoating were chemically labeled with fluorescence through direct coupling by fluoresceinamine. The fluorescence-labeled coating emits green fluorescence at a wavelength of 515 nm upon excitation at a wavelength of 490 nm. A confocal microscope (Olympus FV1200 laser scanning confocal microscope) was used to scan the fluorescence signals of the hydrogels in an area of 400 μm by 400 μm layer by layer throughout the thickness (the distance between each layer was 20 μm). The images were reconstructed in three dimensions using ImageJ to visualize the polymer brush distribution.

### Contact angle measurement

The surfaces of all hydrogels were wiped dry using Kimwipes (Kimberly-Clark Kimtech Kimwipes) before water contact angle tests. Contact angle images were taken and analyzed using a Kruss drop-shape analyzer. All measurements used a 2-μl drop of DI water.

### Preparation of hydrogel bioadhesives

Hydrogels used to prepare hydrogel bioadhesives include PAAM and PVA hydrogels. The PAAM hydrogels can be either copolymerized with HMAAM with the AAM to HMAAM molar ratio of 100:1 or mixed with 2 wt % HA. All hydrogels were coated with a PAA brush on both sides. The coated hydrogels were first washed in water to remove reaction residues, then fixed between two acrylic frames, and dried in an oven at a temperature of 50°C. The dried hydrogels were immersed in an HCl solution (pH = 1) containing 10 wt % EDC and 5 wt % sulfo-NHS for 15 min to introduce NHS ester groups to the PAA brush (*32*) and then immediately placed in a continuous nitrogen flow to dry the water and evaporate HCl.

### Tensile tests of hydrogels

All tests were conducted in the open air and at room temperature. To measure the stress-strain curve of a hydrogel, a hydrogel specimen with a thickness of 1 mm and a stretch area of width 1 cm and height 1 cm was clamped with two grips and mounted on an Instron testing machine (Instron 5943). A tensile force was applied to stretch the hydrogel in its height direction at a rate of 0.5 mm/s until rupture. Force (N) and displacement (mm) were recorded during the tests. The force was converted to stress (Pa) by dividing it by the cross-sectional area in the undeformed state (10^−5^ m^2^). The displacement was converted to strain by dividing it by the height in the undeformed state (10 mm).

### Fracture toughness tests of hydrogels

Pure shear tests were conducted to measure the fracture toughness (*58*). Two samples of the same hydrogels were made (3 cm in length and 5 mm in height): one with a pre-crack of 8 mm along the midline by a sharp razor blade and the other without. All samples were tested individually on an Instron testing machine at a rate of 0.5 mm/s. The pristine samples were used to measure the stress-stretch curve. In the reference state when the sample is not deformed, the height of the sample is *H*; after stretch, the height becomes (1 + ε)*H*, where ε is the strain. The area beneath the stress-stretch curve gives the energy density stored in the hydrogel, *U*(ε) (figs. S19A and S20A). The pre-crack samples were used to measure the critical strain ε_c_ for fracture, where the pre-crack turns into a running crack (fig. S19, B to F). The fracture energy is calculated as Γ = *U*(ε_c_)*H*.

### Adhesion tests

All tests were conducted in the open air and at room temperature. Tissue samples were freshly cut into testing specimens of dimensions 1 cm by 2 cm by 1 mm, washed in PBS, wiped off the water on the tissue surfaces, and stored in a plastic bag to prevent dehydration. Dry bioadhesives were cut into a size of 1 cm by 1 cm and directly placed between two pieces of tissue samples, and gently pressed with fingertips for 5 s. Adhesion tests were performed 20 hours after the initial pressing to ensure equilibrium. For testing adhesion kinetics, adhesion tests were performed at each time point after the initial pressing.

T-peel tests were used to measure adhesion energy (fig. S20A). The back sides of the tissues were glued with stiff polyester films (clear polyester film, McMaster-Carr) with a thickness of 100 μm using a cyanoacrylate instant glue (e.g., Krazy glue). Polyester films restrict the deformation of tissues during peeling. The free ends of tissues were fixed to an Instron testing machine. The peeling rate was fixed at 0.5 mm/s. The peeling force with displacement was recorded (fig. S20B). The adhesion energy was calculated as twice the value of the peeling force at the plateau divided by the width of the sample (*71*).

Lap-shear tests were used to measure shear strength. Two tissue pieces were adhered, and their back sides were glued with two polyester films (fig. S20C). A tensile force was applied to the two ends of the tissues to pull them apart at a rate of 0.5 mm/s until failure. The shear force was recorded during the tests (fig. S20D). The shear strength was determined by dividing the maximum shear force by the adhesion area (*72*).

### Roughness measurement of hydrogel surfaces

Surface roughness mapping on hydrogels was measured by an optical surface profiler (TopMap Micro.View+, Polytec; ZYGO 3D optical profilometer) using white light interferometry. A 5× lens was used to measure hydrogels with no coating and nanocoating, and a 50× lens was used to measure hydrogels with microcoating. Raw data were processed to produce two-dimensional contour profiles.

### In vitro biocompatibility test

PVA hydrogels were coated with PAA and PMPC brushes, sectioned into 4 mm–by–4 mm squares with thicknesses of 300 μm, sterilized by UV irradiation, and sequentially washed three times with sterilized DI water. PVA hydrogels with no coating were used as a control. Sterile samples were then placed into each well of a 24-well plate seeded with HEK293T cells at a density of 5 × 10^4^ cells per well. After 24 hours of incubation in 2 ml of Dulbecco’s modified Eagle’s medium (DMEM) at 37°C with 5% CO_2_, cell viability was determined with a live/dead fluorescent viability dye cocktail (Thermo Fisher Scientific, catalog no. R37601) following the manufacturer’s instructions. The stained cells were then imaged using a Nikon Eclipse Ts2 inverted epifluorescence microscope with the fluorescein isothiocyanate (FITC) and red fluorescent protein (RFP) filters for live and dead cells, respectively. The resulting images were analyzed using ImageJ to quantify cell viability.

### In vivo adhesion tests

All animal procedures were approved by the Massachusetts General Hospital (MGH) Institutional Animal Care and Use Committee (protocol number 2005N000306) and performed in compliance with relevant ethical regulations. Male C57BL/6 mice of 8 to 12 weeks of age were purchased from the Jackson Laboratory. Animals received buprenorphine analgesia (0.05 mg/kg, intraperitoneally) before the procedure. The mice were anesthetized with isoflurane 2% and mechanically ventilated. A left thoracotomy was carried out to access the heart. The PAAM bioadhesive was placed on the free left ventricular wall. The ribs and the skin were closed using 5.0 sutures (Ethicon). The animals were awakened and extubated when breathing independently of the ventilator. Buprenorphine was given twice daily, 3 days postprocedure. After implantation for 7 days, we performed the same procedure to expose the heart and examine the patch. If the patch lost adhesion and moved elsewhere, we further opened the entire chest to locate the patch. After that, the mice were euthanized by CO_2_ inhalation.

### Hydrogel implantation and retrieval

All animal procedures were approved by the Massachusetts Institute of Technology (MIT) Committee on Animal Care (protocol number 0620-045-23) and supervised by the MIT Division of Comparative Medicine veterinary staff. Male C57BL/6 mice of 6 to 8 weeks of age were obtained from the Jackson Laboratory and used in the experiments. Animals were anesthetized with 3% isoflurane in oxygen. To prepare for surgery, the mice’s eyes were covered with ocular lubricant (Optixcare Eye Lube). The mice’s abdomens were shaved and sterilized using betadine and isopropanol and were administered with 60 μl of Buprenorphine SR-LAB (ZooPharm) (1 mg/kg) and 100 μl of 0.9% saline subcutaneously.

All surgical tools were sterilized by autoclaves, and all hydrogels were sterilized by UV light for 2 hours. Hydrogel implants included PVA hydrogels with no coating, PMPC nanocoating, and PMPC microcoating, as well as alginate hydrogels and PAAM hydrogels with PMPC microcoatings. Hydrogel implants were made by cutting into a disc shape of 8 mm in diameter using a biopsy punch (Integra Miltex Standard Biopsy Punches, 8 mm in diameter). The thicknesses of hydrogel implants were 350 μm for PVA hydrogels, 650 μm for alginate hydrogels, and 440 μm for PAAM hydrogels.

A small incision along the midline of the abdomen was made and followed by blunt dissection to expose the peritoneal lining. An incision was made along the linea alba, and hydrogels were inserted in the abdomen, away from the fat pad. Each mouse received one hydrogel. The incision was closed using absorbable Vicryl sutures (Ethicon Inc.). The skin was closed over the incision using wound clips (EZ Clip Wound Closures).

After 40 days, all implanted hydrogels were due for retrieval. The 40-day implantation period is sufficient to trigger the FBR. During retrieval, mice were euthanized by CO_2_ inhalation and cervical dislocation. We opened the peritoneal cavity and located the implanted hydrogels. Care was taken to include any fibrotic tissue when retrieving the hydrogels. The retrieved hydrogels were transferred and stored in PBS solutions for further histology analysis.

### Histology analysis

Retrieved hydrogels were fixed using 10% formalin for 1 hour at room temperature and immersed in a 2 wt % agarose solution (Agarose, ultralow gelling temperature; Sigma-Aldrich, A2576) at 40°C. The hydrogel implants were embedded inside the agarose hydrogels upon gelation when the temperature was cooled down to room temperature. This procedure prevented the loss of fibrotic tissues on the hydrogel surfaces during the histology sectioning process.

Histology analysis was performed at the Hope Babette Tang Histology Facility at the Koch Institute at MIT. Hydrogel samples were dehydrated in 70% ethanol, embedded in a paraffin block, and sectioned in the hydrogel thickness direction at three different locations to reveal the thickness of the fibrotic capsule. The sectioned samples were further processed with H&E staining and Masson’s trichrome staining according to standard histological methods.

### Hydroxyproline assay to quantify the concentration of deposited collagen

Retrieved hydrogels were immersed in a 1:1 mixture of water and 37% hydrochloric acid in a glass vial, screwed the cap tightly, and further sealed by wrapping the cap with Teflon tape. The solution was hydrolyzed at 120°C for 12 hours and subsequently analyzed the collagen concentration using a hydroxyproline collagen assay kit (Chondrex, catalog no. 6017) according to the manufacturer’s instructions.

### Statistical analysis

Details of the sample size and appropriate statistical tests are included in the figure captions. All in vitro experiments were repeated at least five times unless specified. For the histology analysis, the data were analyzed for statistical significance by unpaired, two-tailed Student’s *t* test, as implemented in GraphPad Prism 7. *P* values are indicated in the figures.

## Supplementary Materials

### The PDF file includes:

Supplementary Text Figs. S1 to S20 Legends for movies S1 to S3 Competing interests statement References
