# Supplementary material for: A modular hydrogel system with independent control of bioadhesion, fibrosis, and stiffness
Source: Sci Adv. 2026 Jun 24;12(26):eaee3894. doi: 10.1126/sciadv.aee3894 (PMC13292947; doi:10.1126/sciadv.aee3894)
Supplement: Supplementary file 1 — Supplementary Text Figs. S1 to S20 Legends for movies S1 to S3 Competing interests statement References [file sciadv.aee3894_sm.pdf]

Science Advances
AAAS

Supplementary Materials for

A modular hydrogel system with independent control of bioadhesion, fibrosis, and stiffness

Jiawei Yang *et al.*

Corresponding author: Jiawei Yang, jyang11@wpi.edu; Daniel G. Anderson, dgander@mit.edu

Sci. Adv. **12**, eaee3894 (2026)
DOI: 10.1126/sciadv.aee3894

The PDF file includes:

Supplementary Text
Figs. S1 to S20
Legends for movies S1 to S3
Competing interests statement
References

Other Supplementary Material for this manuscript includes the following:

Movies S1 to S3

Supplementary Text

Estimation of the occupied area of a bromide atom

Because each bromine atom can grow one polymer brush, the occupied area of a bromine atom is the same as the polymer brush graft area (*74*). A PVA hydrogel is made of PVA chains crosslinked by hydrogen bonds. Each PVA repeat unit bears one OH group. In a PVA chain consisting of $l + m$ units of OH groups, where m units of OH groups are chemically converted to tertiary bromide and l units of OH groups remain. Define the degree of modification of OH groups as $r_1 = m/(l + m)$, then the degree of unmodified units is $1 - r_1$. The combined modified and unmodified units constitute a new PVA repeated unit. XPS analysis shows that the Br atomic concentration among C, O, and Br is 4.41%. In this newly constructed repeat unit,

$$\text{Br atom\%} = \frac{r_1}{9r_1 + 3(1 - r_1)} = 0.0441,$$

which gives $r_1 = 0.18$, indicating that 18% of PVA units are chemically modified. Therefore $l/m = 4$ (Fig. S4).

Next, we calculate the occupied area for a bromine atom. The volume of each monomeric unit can be estimated by $V = M/A\rho$ (*75*), where M is the molecular weight, A is the Avogadro constant (6×10^{23} 1/mol), and ρ is the density. For PVA, $M = 44$ g/mol, $\rho = 1.19$ g/ml, and $V_{\text{PVA}} = 0.062\ \text{nm}^3$; for bromine-modified PVA, $M = 193$ g/mol and $\rho = 1.19$ g/ml, $V_{\text{PVA-Br}} = 0.27\ \text{nm}^3$. The total volume of a new repeat unit is

$$V_{\text{tot}} = (1 - r_1)V_{\text{PVA}} + r_1 V_{\text{PVA-Br}} = 0.1\ \text{nm}^3$$

Therefore, 1.8 bromine atoms occupy a volume of 1 nm^3, and each bromine atom occupies an area of $\sigma = (1/1.8)^{2/3} = 0.68\ \text{nm}^2$.

Thickness control of polymer coating

The thickness of the polymer coating is controlled by the amount of added sacrificial initiators in the reaction solution to tune the monomer-to-initiator ratio, which is also the degree of polymerization N. We assume a well-controlled ATRP so that polymer chains generated on the surface of hydrogels and in the solution have the same N. Because the polymer brush thickness linearly relates N in the contact and high-density brush conformations (*76-78*), varying N changes the thickness.

According to SEM imaging, we assume the bromine modification mostly takes place within a depth of ~1 μm and neglect those beyond this depth (Fig. S2C). The surface area of the dry hydrogel is ~10 mm^2 and the bromine-modified volume is ~0.01 mm^3. Given the volumetric density of bromine atoms of 1.8 atoms/nm^3, the bromine is ~10^{-8} mol fixed in the hydrogel. Note that this is an overestimation as the bromine concentration decays away from the surface. The reaction solution is about 1 ml, and thus the fixed bromine (ATRP initiator) concentration is ~10^{-5} M. In our experiments, the sacrificial initiators added are 0.1 M and 0.001 M, at least two orders of magnitude higher than the fixed initiators, hence, the concentration of ATRP initiators is determined by the sacrificial initiators. The monomer concentration is typically ~1 M, thus $N \sim 10$, 10^3, and 10^5 (with no sacrificial initiator).

Estimation of polymer coating thickness

For a PVA hydrogel coated by a polymer brush, the spacing between anchored polymers is $s = (1/1.8)^{1/3} = 0.8$ nm. The size of a polymer chain in the unperturbed state is estimated by the Flory radius (*79*), $r \sim aN^{1/2}$, where $a = V^{1/3}$, the monomer size. If $r \ll s$, the anchored polymers are dilute and do not contact, and the thickness of polymer coating $t \sim r$; if $r \gg s$, the anchored

polymers contact and are closely packed. The steric confinement from the neighbor polymers stretches the polymer to reduce interaction. In the limiting case where the polymer is fully stretched, the thickness of the polymer coating is the contour length of the polymer brush, $t = aN$.

For acrylic acid, $M = 72$ g/mol and $\rho = 1.05$ g/ml, $a = 0.5$ nm. Therefore, the PAA polymer size in the unperturbed state is $r = 1.6$ nm, 15.8 nm, and 158 nm for nano-coating ($N \sim 10$), 1 microscale coating ($N \sim 10^3$), and 10 microscale coating ($N \sim 10^5$). Note that these values are all bigger than s, and hence the polymer brushes are all in the contact brush conformation.

In the Alexander-de Gennes scaling analysis for a contact polymer brush (*76, 77*), t linearly scales with N. For the primary estimation, we simply use the contour length of the polymer chain to estimate the thickness, and we obtain $t \sim 5$ nm, ~0.5 µm, and ~50 µm, which fairly agree with the experimental observations.

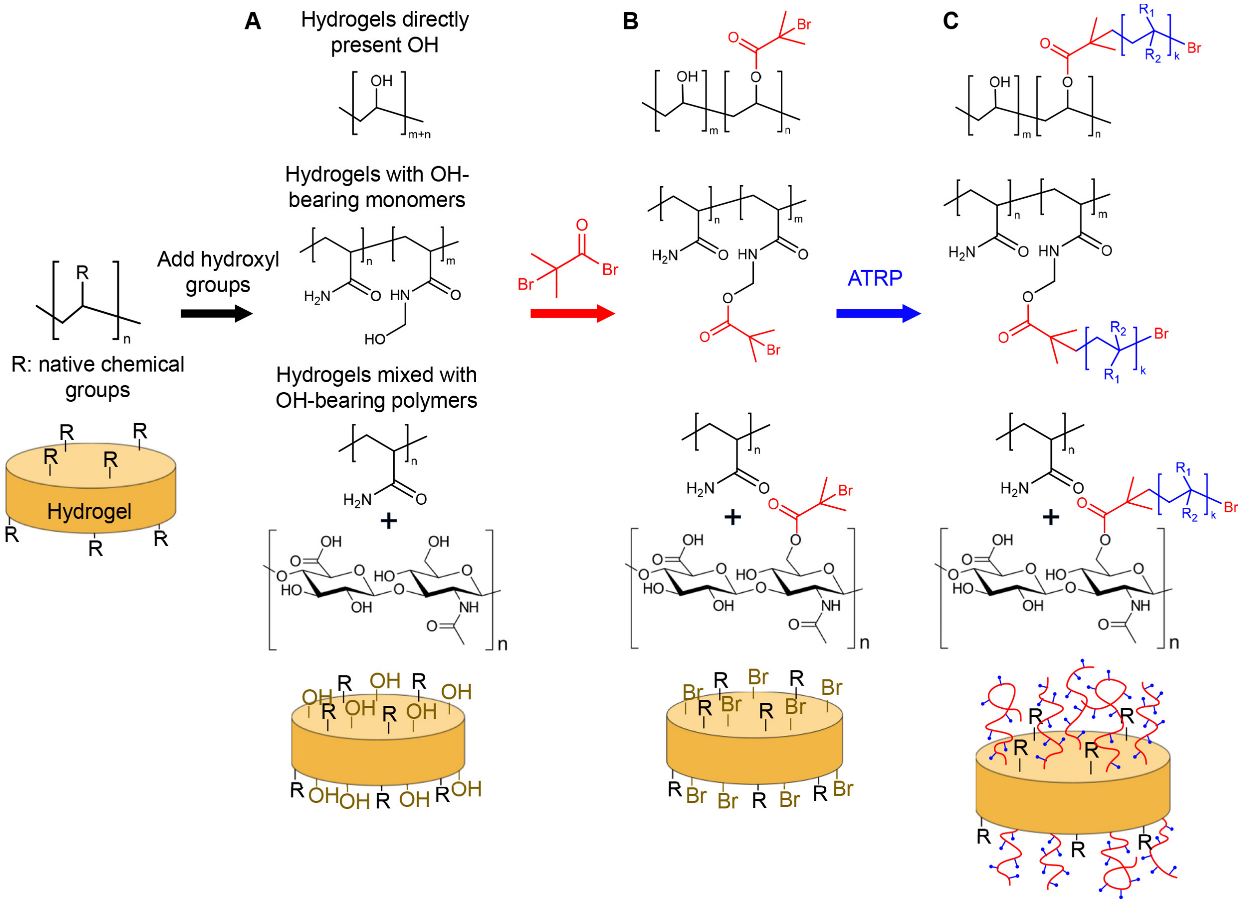

Fig. S1.

Polymer brush coating process. A hydrogel is first added with OH groups by either physical or chemical means. **(A)** Three representative examples used in this work are PVA hydrogels that directly present OH groups, PAAM hydrogels chemically incorporated with OH-bearing HMAAM monomeric units, and PAAM hydrogels physically mixed with OH-bearing HA polymers. **(B)** The OH groups on the surfaces of hydrogels are subsequently converted to tertiary bromides by reacting with BriBr. **(C)** The polymer brush coating covalently bonded to the hydrogel is realized by growing polymers from the surface-anchored tertiary bromides by ATRP.

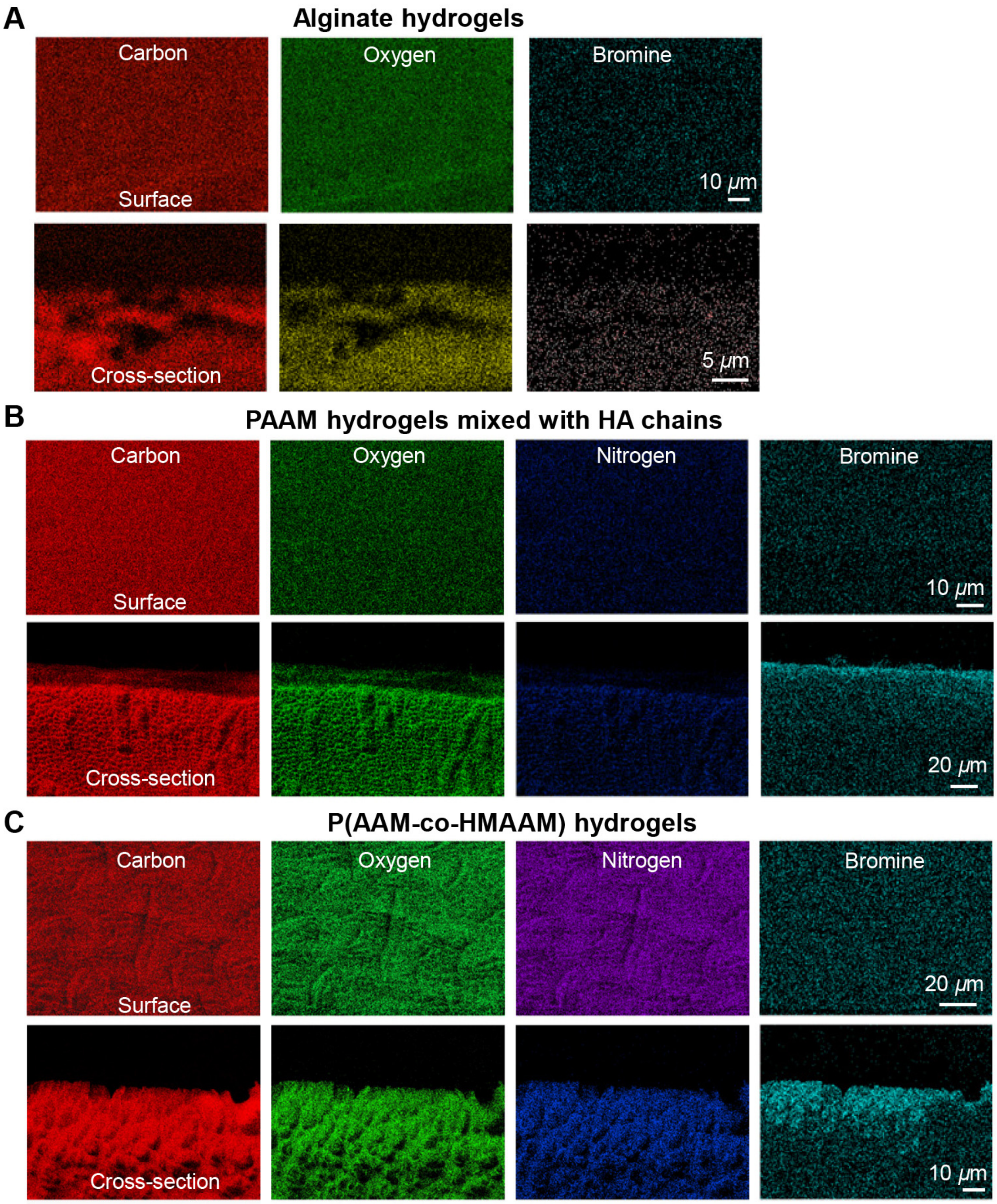

Fig. S2.

Element distribution in tertiary-bromide-modified hydrogels. (A) Alginate hydrogels. **(B)** PAAM hydrogels mixed with HA chains. **(C)** PAAM hydrogels incorporated with HMAAM units.

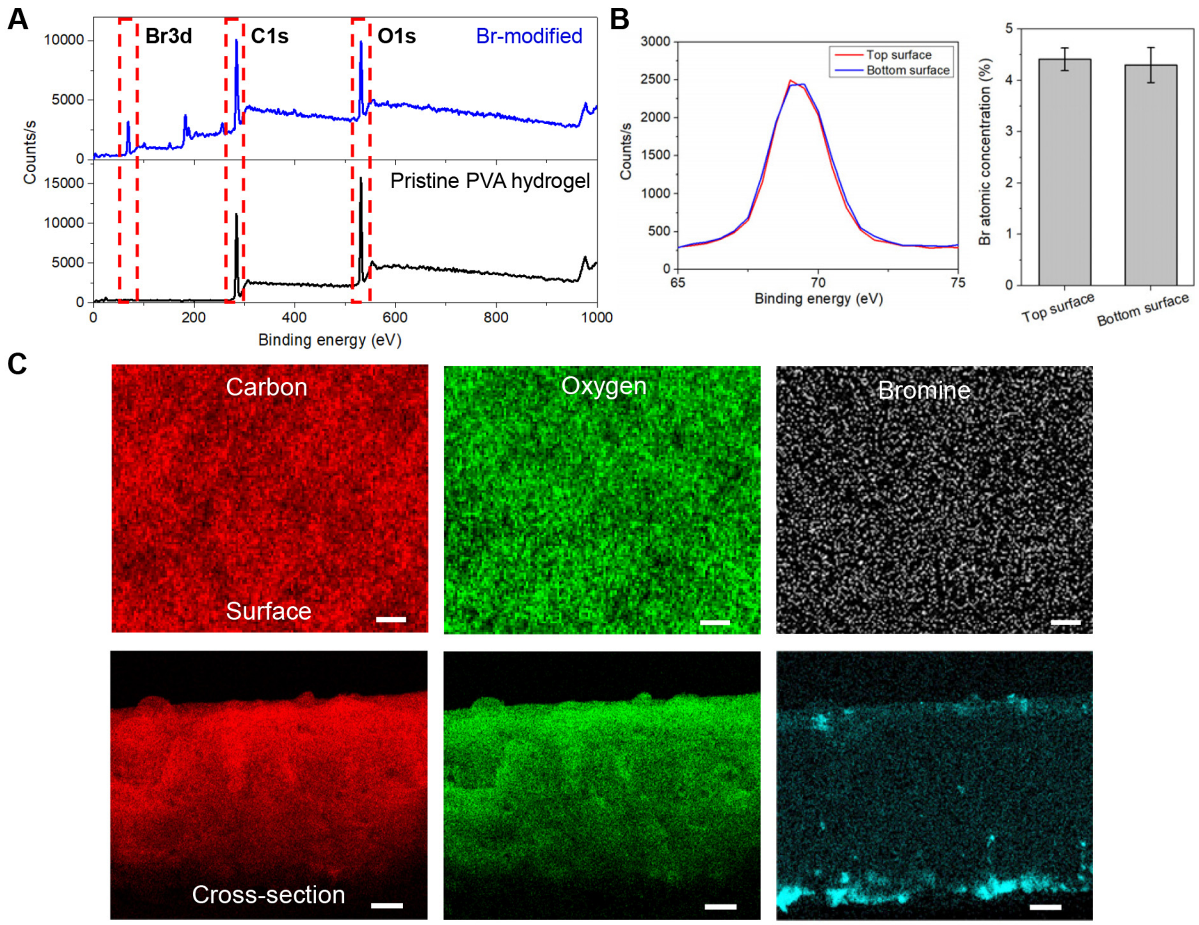

Fig. S3.

Element analysis for the tertiary-bromide-modified PVA hydrogels. (A) XPS exhibits a new Br3d peak in Br-modified PVA hydrogels. **(B)** Bromine atomic concentration is the same on both surfaces, which is about 4.4%. **(C)** SEM-EDS illustrates the atom distribution in the hydrogel. On the surface, carbon, oxygen, and bromine atoms are uniformly distributed. At the cross-section, carbon and oxygen atoms remain uniform, but bromine atoms are highly localized near the surfaces and sparsely distributed in the bulk. Scalar bars: 10 µm.

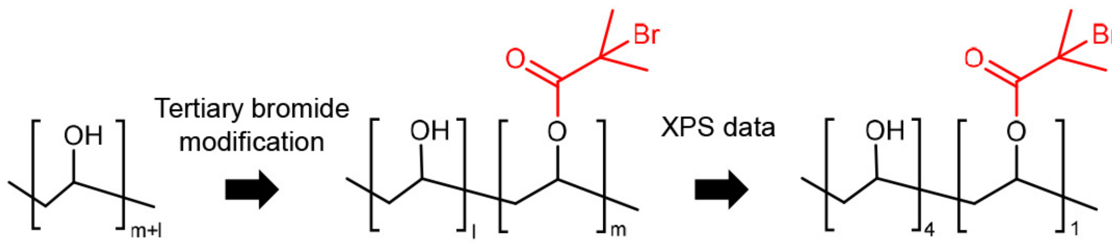

Fig. S4.

Degree of Br modification. As estimated from the XPS data, the ratio of modified and unmodified PVA units is 4.

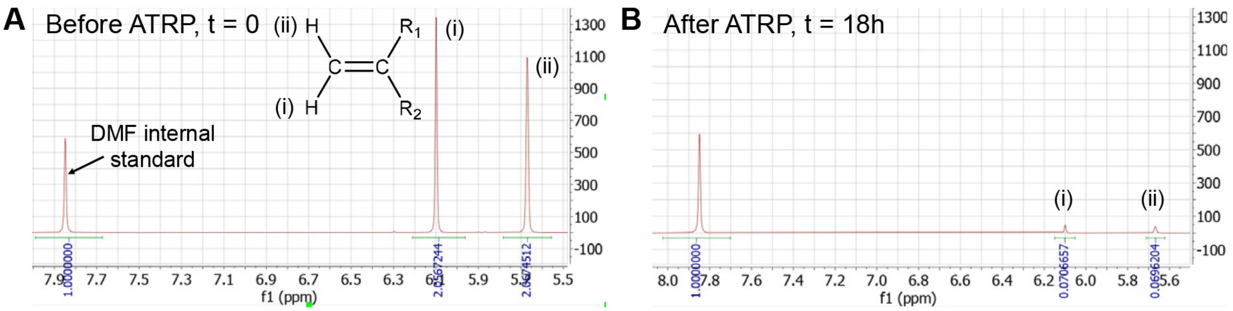

C

	0 h	6 h	18 h
AA	0	46.7 %	49.5 %
MPC	0	97.8 %	100 %
NHS-acrylate	0	78 %	92 %

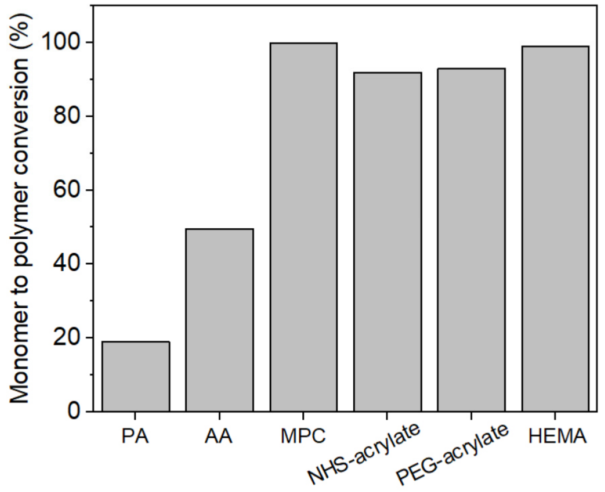

Fig. S5.

Proton NMR characterization of ATRP. (A) Before ATRP (t = 0), a monomer (MPC in this case) has two signature peaks of protons adjacent to the double bond, labeled as (i) and (ii). **(B)** After ATRP, monomers convert to polymers, where double bonds in the monomers become single bonds, which reduces the (i) and (ii) peaks. The conversion is calculated as the peak area difference of the protons before and after ATRP divided by the peak area before ATRP. **(C)** Different monomers show different ATRP kinetics. **(D)** Monomer to polymer conversion for several types of monomers after 18 h ATRP.

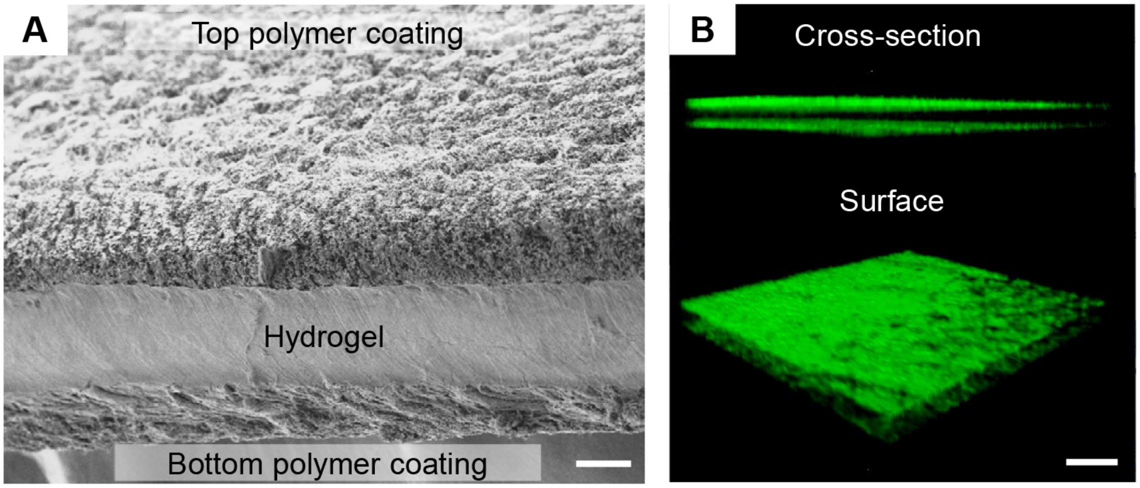

Fig. S6.

Cross-sectional view of the hydrogel with 40-micron PAA brush coating. (A) The SEM image shows a sandwich structure of the center PVA hydrogel and the top and bottom polymer brush coating layers. **(B)** Confocal microscopic images show that the fluorescence-labeled PAA brush coating is localized near the surfaces of hydrogels but not inside. Scale bars: 50 µm.

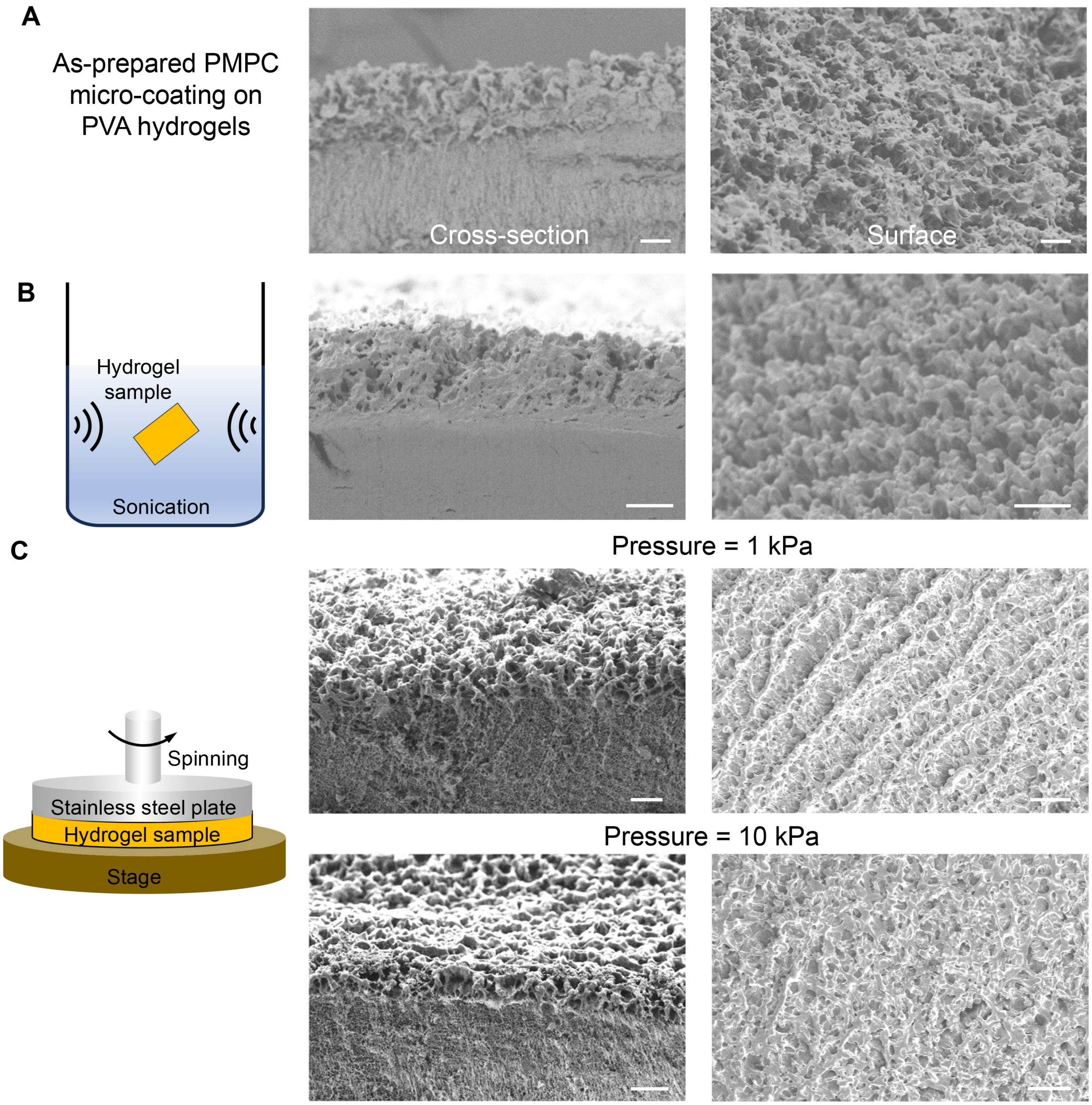

Fig. S7.

Stability tests of the PMPC micro-coating. SEM images of PVA hydrogels with the PMPC micro-coating. **(A)** As prepared. **(B)** Sonication for 30 min. **(C)** Wear against a metal plate under pressures of 1 kPa and 10 kPa for 30 min. Scale bars: 40 µm.

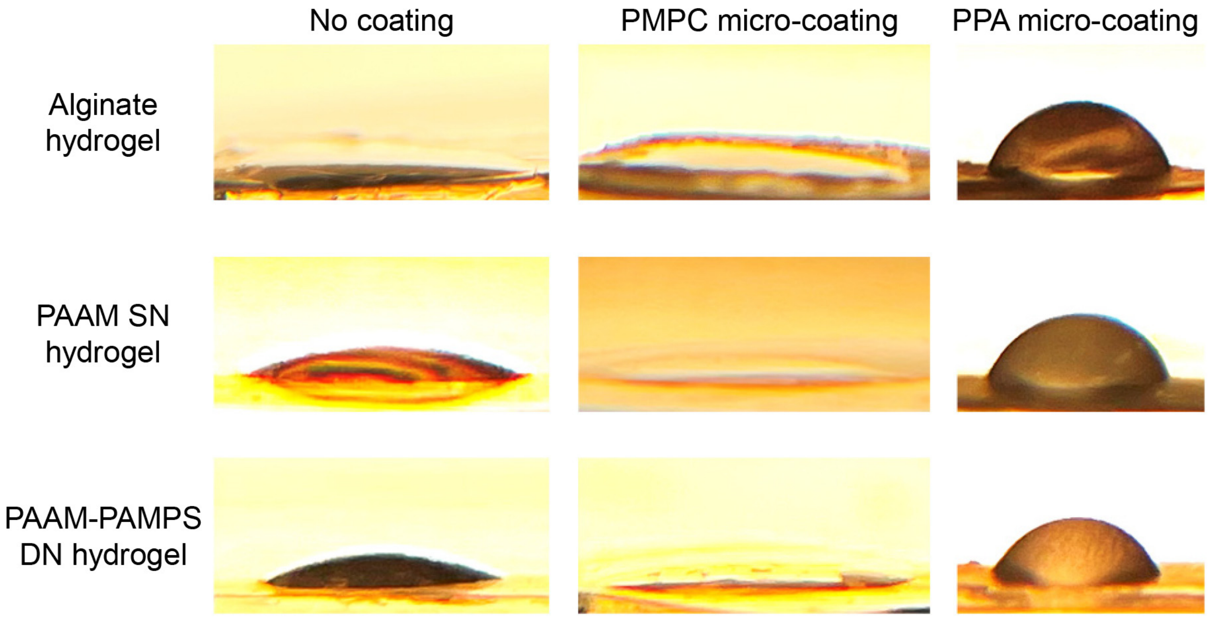

Fig. S8.

Optical images of water contact angle on various hydrogel substrates with the PMPC and PPA micro-coatings. The PMPC micro-coating shows that the water completely wets the surface, while the PPA micro-coating shows that the water beads up.

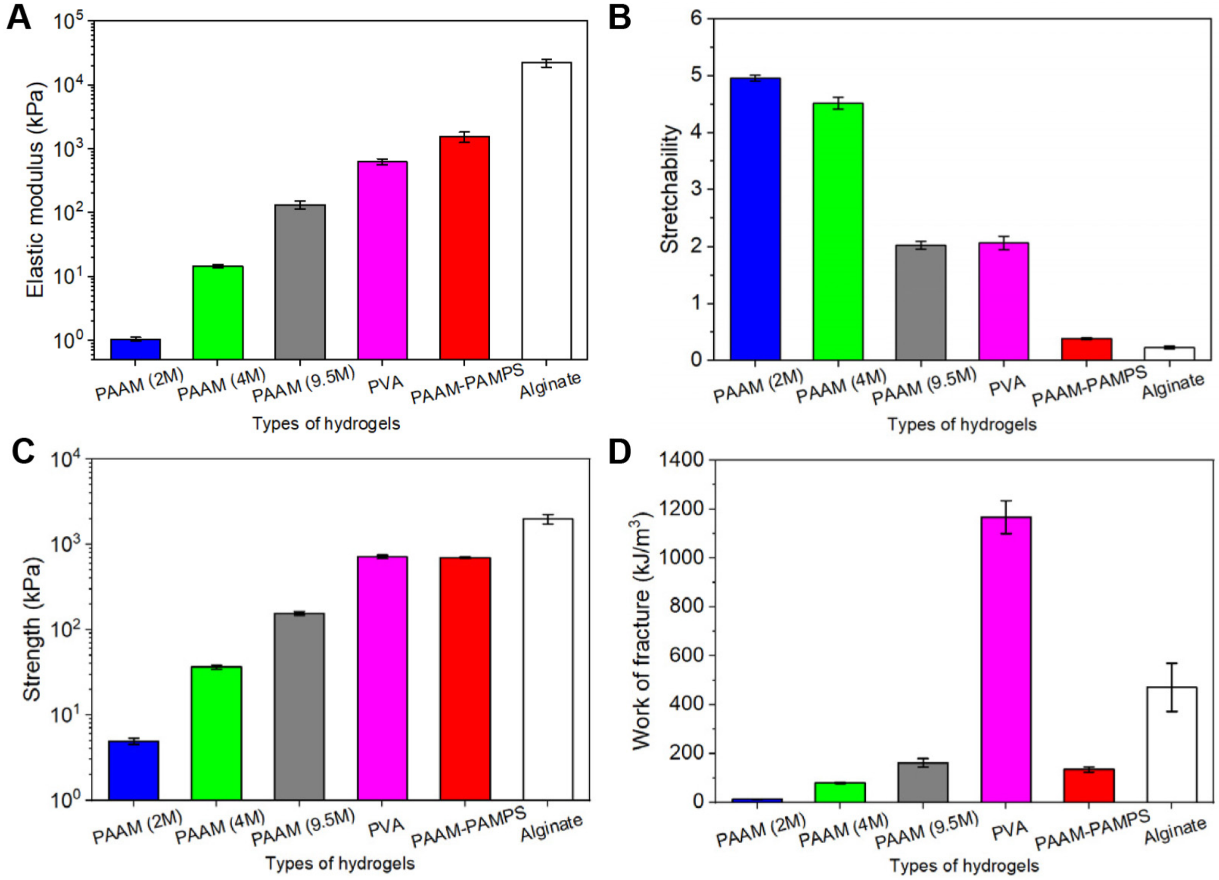

Fig. S9.

Comparison of the mechanical properties of various hydrogels with the PMPC micro-coating. Four types of hydrogels of distinct chemistries and network topologies are compared. **(A)** Elastic modulus varies by 4 orders of magnitude. **(B)** Stretchability varies by 10 times. **(C)** Strength varies by 2 orders of magnitude. **(D)** Work of fracture varies by 3 orders of magnitude.

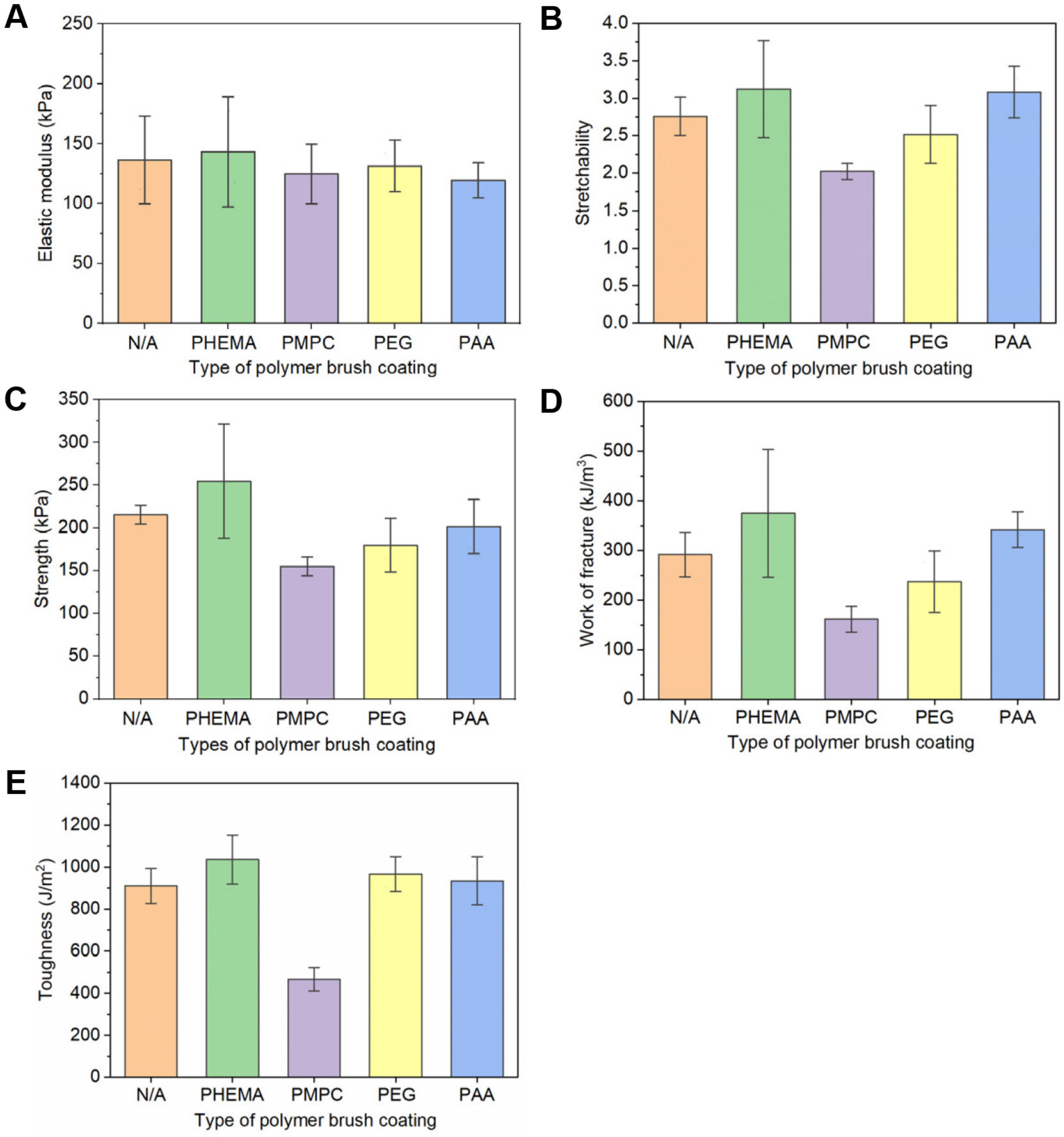

Fig. S10.

Comparison of the mechanical properties of PAAM hydrogels with various micro-coatings. **(A)** Elastic moduli, **(B)** Stretchability, **(C)** Strengths, **(D)** Work of fracture, and **(E)** Fracture toughness.

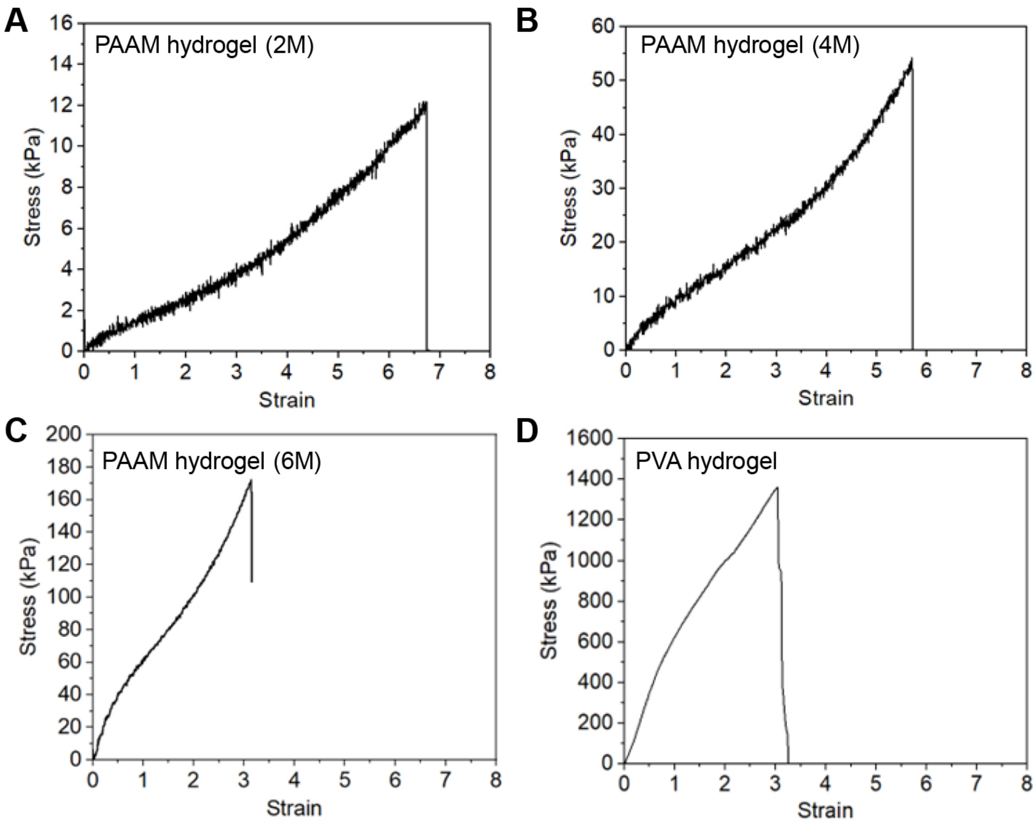

Fig. S11.

Stress-strain curves of PAAM and PVA hydrogels with the PAA micro-coating. (A)-(C), Increasing the polymer concentration stiffens the hydrogel. **(D)** Further increasing stiffness using semi-crystalline PVA hydrogels. The elastic moduli of the four hydrogels span the full range of soft tissues (1 kPa – 1 MPa).

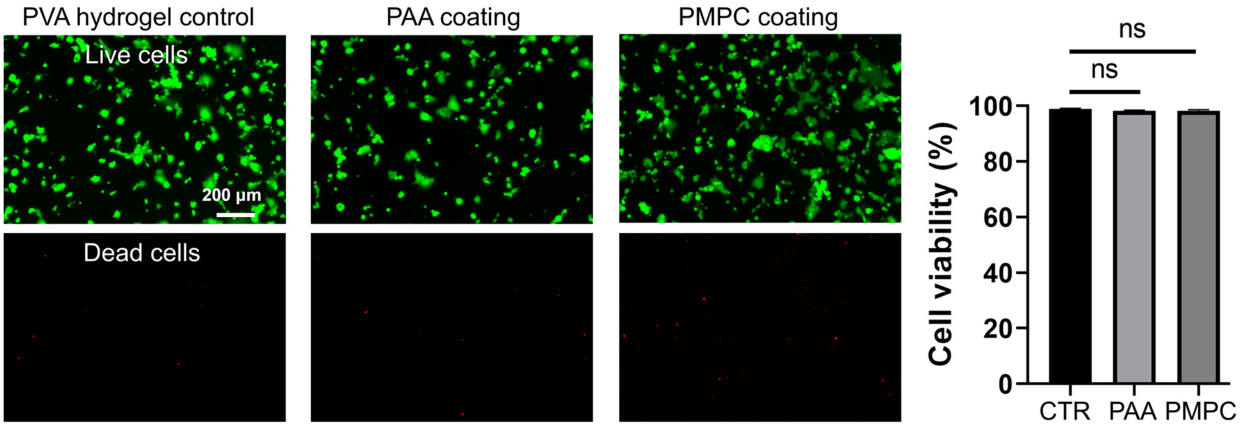

Fig. S12.

In vitro biocompatibility tests for PVA hydrogels with PAA and PMPC coatings. Tests are performed in a live/dead assay of HEK 293T cells after 24 hours of culture (live cells are stained green and dead cells are stained red). Cell viability in the three hydrogel groups is similar. Statistical analysis: two-tailed Student's t tests that compare CTR vs. PAA-coating and CTR vs. PMPC-coating.

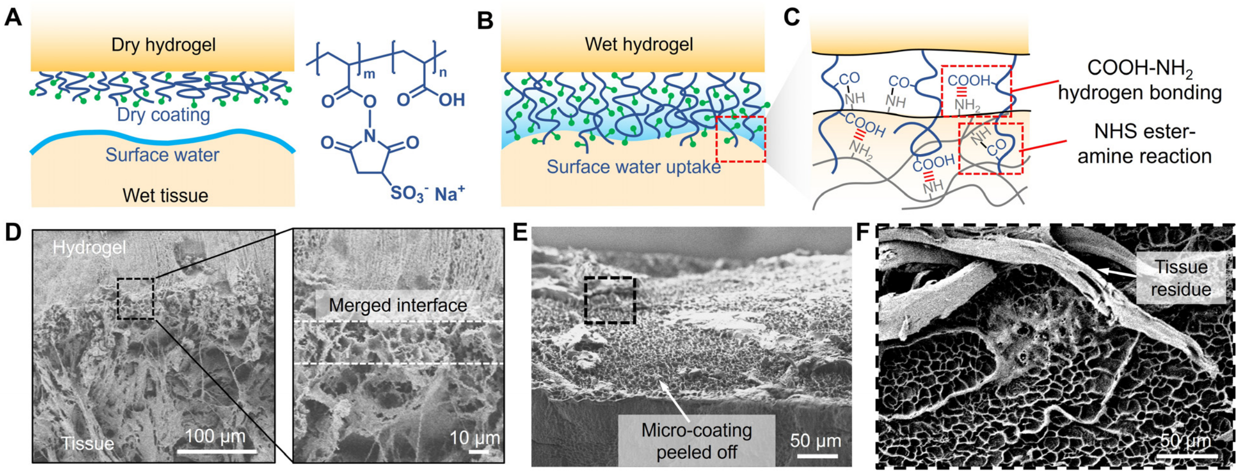

Fig. S13.

Adhesion and separation of bioadhesives and tissues. (A) A hydrogel is coated with a P(AA-co-NHS ester) brush, then completely dried, and applied onto a wet tissue. **(B)** The dry hydrogel and the brush absorb the surface water, hydrate, and swell. **(C)** During this process, carboxylic acid groups and NHS ester groups on the P(AA-co-NHS ester) brush form intermolecular hydrogen bonds and peptide bonds with amine groups on the tissue. **(D)** The SEM image shows the micro-coating merges with the tissue. **(E)** Upon separation, the micro-coating is peeled off from the hydrogel surface, and **(F)** the tissue residue is seen on the hydrogel surface.

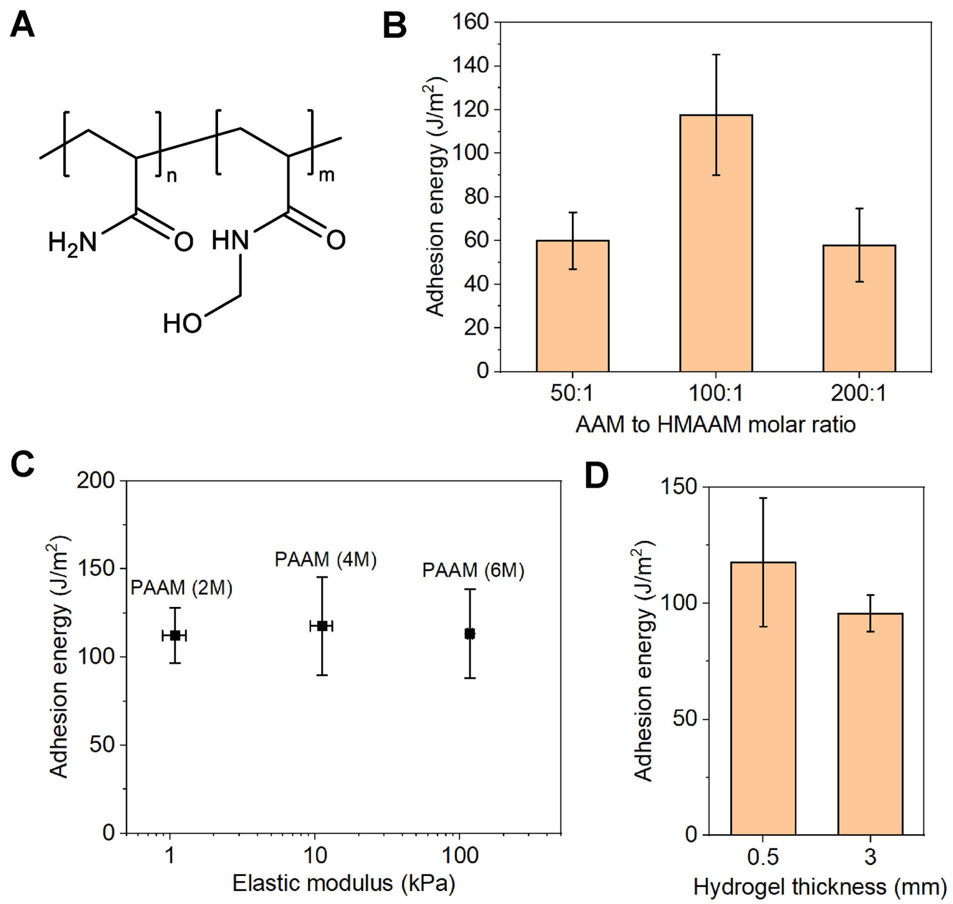

Fig. S14.

Design and characterization of the PAAM bioadhesives. (A) The PAAM hydrogel is made by copolymerization of AAM and HMAAM monomers. **(B)** Optimal adhesion performance is achieved by tuning the AAM to HMAAM molar ratio to 100:1. Adhesion energy is independent of **(C)** the stiffness of the hydrogel, and **(D)** the thickness of the hydrogel.

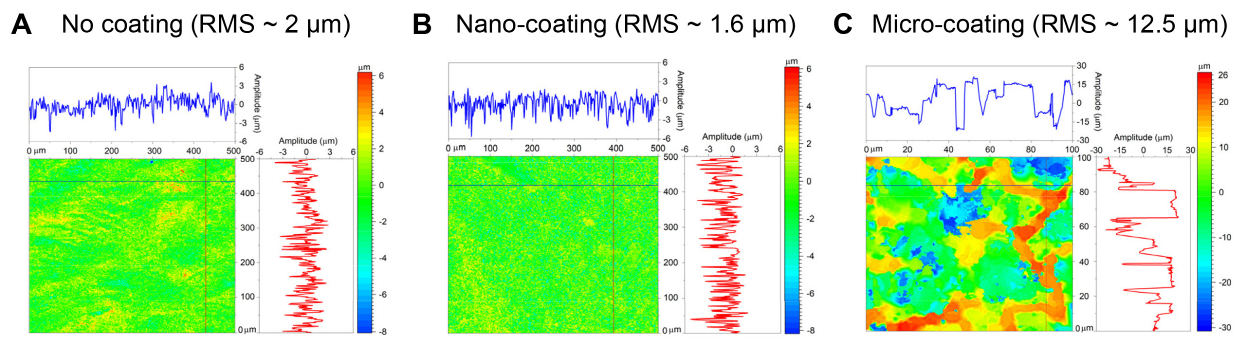

Fig. S15.

Characterization of surface roughness. (A) A pure PVA hydrogel shows a root mean square (RMS) of surface roughness of ~2 µm, while a PVA hydrogel with **(B)** nano-coating exhibits a smoother surface with RMS of ~1.6 µm, and **(C)** micro-coating exhibits a much rougher surface with RMS of ~12.5 µm.

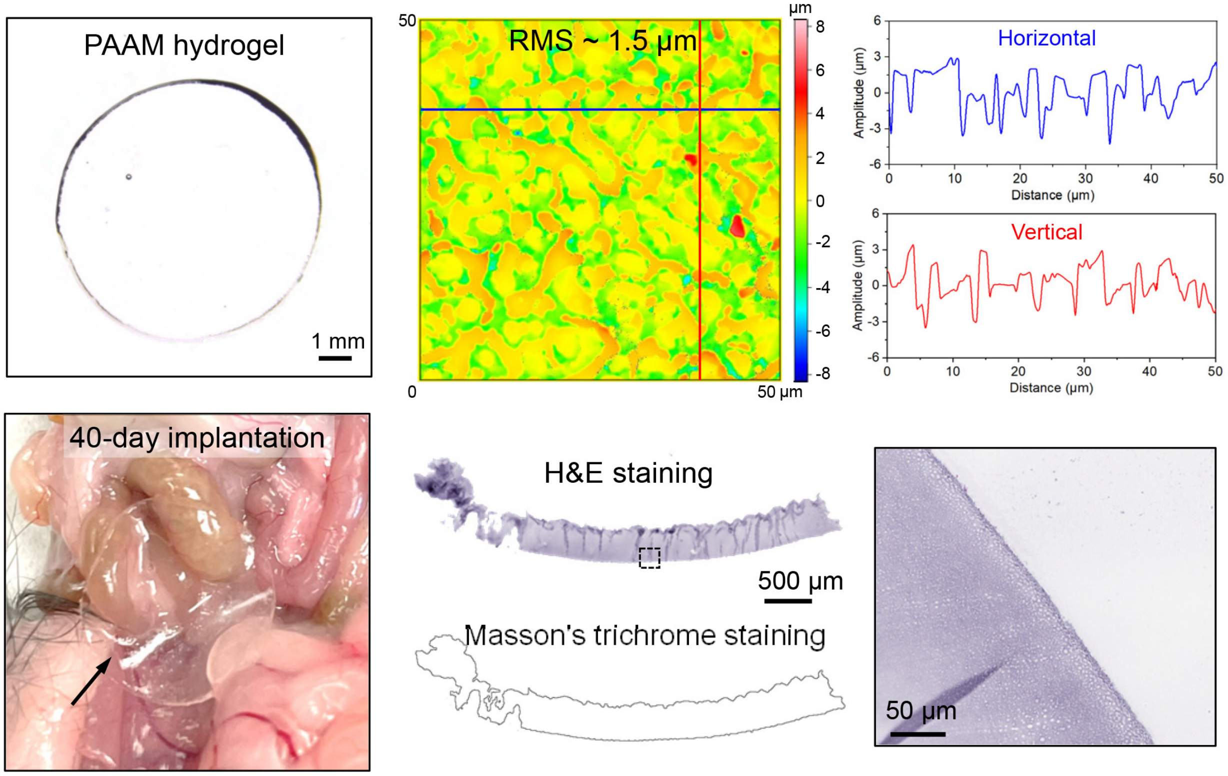

Fig. S16.

Surface roughness and histology analysis for PAAM hydrogels with the PMPC coating. PAAM hydrogels exhibit an RMS of surface roughness of ~1.5 μm, similar to that of the PVA hydrogel with nano-coating. They suppress fibrosis after a 40-day implantation in the IP space of immune-competent C57BL/6 mice, as validated by both visual inspection and histological H&E and Masson's trichrome staining. The hydrogels remain intact upon retrieval. Black arrows indicate the hydrogel implants.

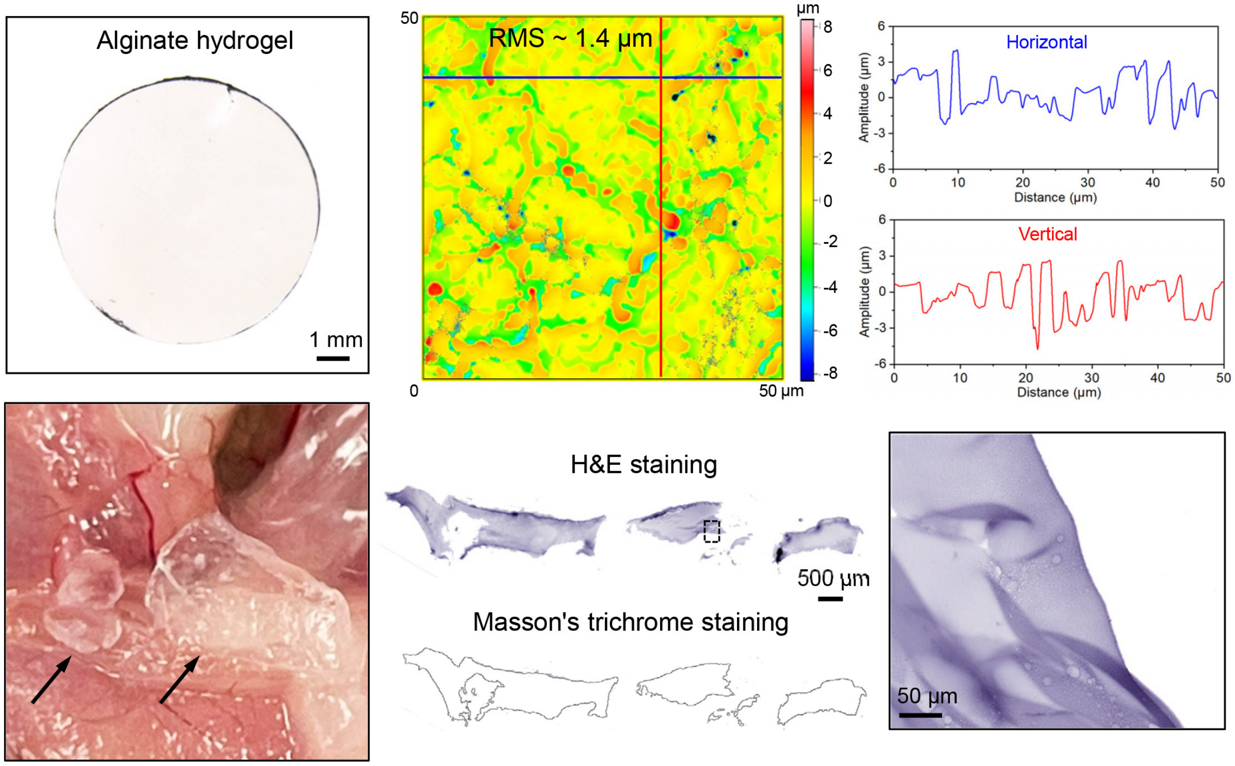

Fig. S17.

Surface roughness and histology analysis for alginate hydrogels with the PMPC coating. Alginate hydrogels exhibit an RMS of surface roughness of ~1.4 µm, similar to that of the PVA hydrogel with nano-coating. They suppress fibrosis after a 40-day implantation in the IP space of immune-competent C57BL/6 mice, as validated by both visual inspection and histological H&E and Masson's trichrome staining. However, they fracture in the body due to weak mechanical properties. Black arrows indicate the hydrogel implants.

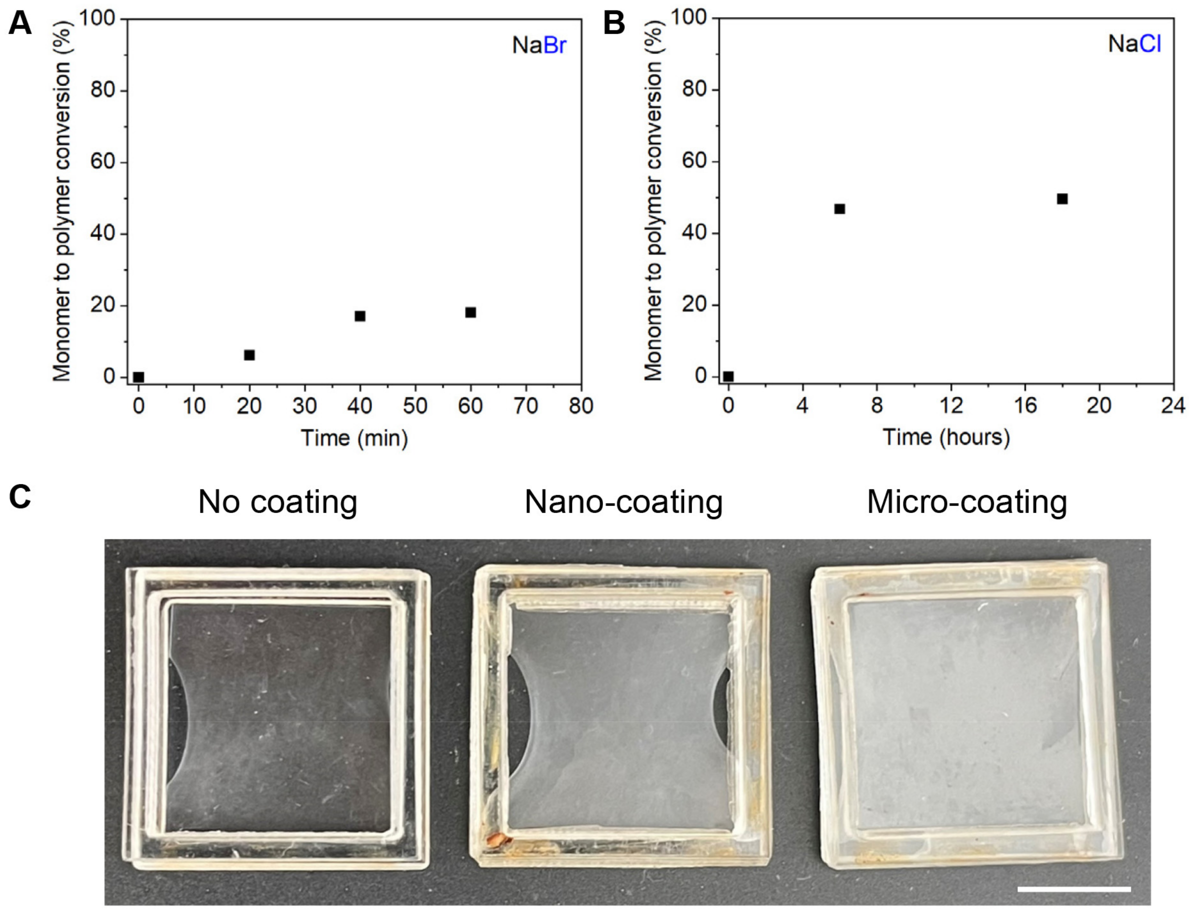

Fig. S18.

Control PAA coating thickness using different salts. Monomer-to-polymer conversion kinetics using **(A)** NaBr and **(B)** NaCl, where the former gives a conversion of ~17% and the latter gives a conversion of ~50%. **(C)** Optical images of PVA hydrogels with no coating, PAA nano-coating, and PAA micro-coating, dried by fixing them in acrylic frames. Scale bar: 1 cm.

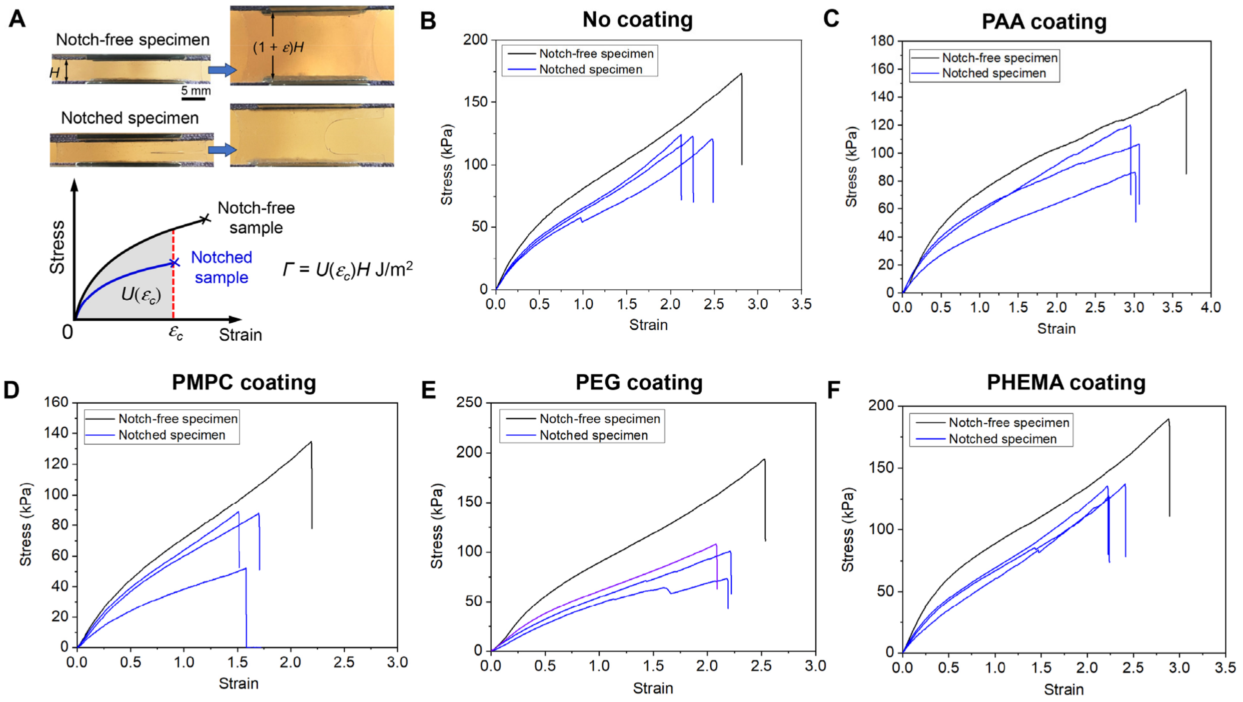

Fig. S19.

Fracture toughness tests of PAAM hydrogels with a variety of polymer brush micro-coatings. **(A)** Pure shear test. Two hydrogels are prepared: one with a pre-crack along the midline and the other without. Two hydrogels are stretched until fracture, where the notch-free hydrogel measures the stress-strain curve, and the notched hydrogel measures the critical strain ε_c for fracture. The fracture energy is calculated as $\Gamma = U(\varepsilon_c)H$, where U is the strain energy density. Stress-strain curves of **(B)** uncoated PAAM hydrogels, and PAAM hydrogels with **(C)** PAA, **(D)** PMPC, **(E)** PEG, and **(F)** PHEMA micro-coatings.

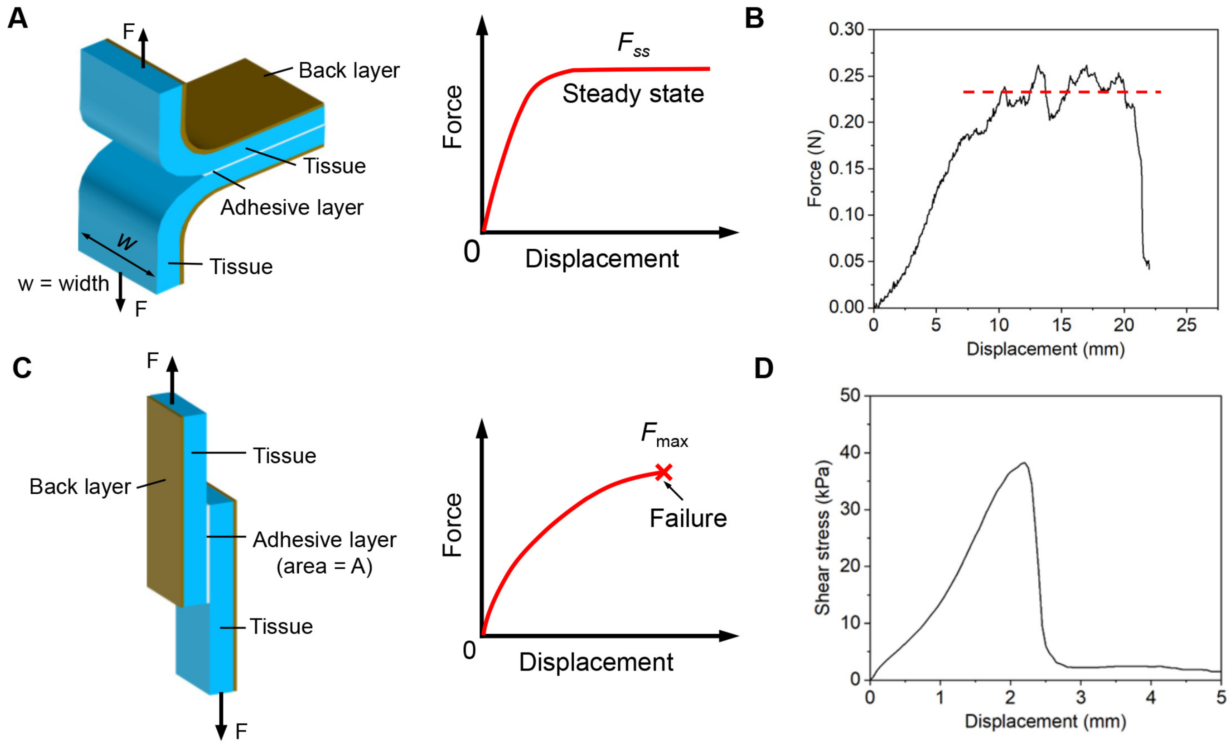

Fig. S20.

Adhesion tests. (A) T-peel tests are used to measure adhesion energy. A force is applied to peel the two adhered hydrogels apart, and the steady state force F_{ss} is measured. **(B)** Peel force as a function of displacement. Adhesion energy relates to the steady state force by $\Gamma = 2F_{ss}$ /w. **(C)** Lap-shear tests are used to measure adhesion shear strength. A force is applied to shear the two adhered hydrogels apart, and the maximum force of failure is measured. **(D)** Shear stress as a function of displacement. Adhesion shear strength relates to the failure force by $\tau_{max} = F_{max}/A$.

Movie S1.

In vivo bioadhesion on a beating heart. The movie shows a surgical procedure of adhering a PAAM bioadhesive to a fast-beating heart without pressing. The adhesion is established within seconds.

Movie S2.

The bioadhesive with micro-coating maintains adhesion after 7-day implantation. The movie shows that the PAAM bioadhesive with micro-coating remains strong and stable on the heart despite the attempt to peel it off.

Movie S3.

The bioadhesive with nano-coating loses adhesion after 7-day implantation. The movie shows that the PAAM bioadhesive with nano-coating has lost adhesion on the heart and migrated to the lung due to weak and unstable adhesion in the body.

2023 Competing Interests Disclosure

From FY 2018 to the present, Dr. Robert Langer receives licensing fees (to patents in which he was an inventor on) from, invested in, consults (or was on Scientific Advisory Boards or Boards of Directors) for, lectured (and received a fee), or conducts sponsored research at MIT for which he was not paid for the following entities:

1. 611 Therapeutics
2. Abpro International;
3. Acorda (Formerly Civitas Therapeutics);
4. Alfred University;
5. Aleph Farms;
6. Alivio Therapeutics;
7. Alkermes;
8. Allevi;
9. Allurion;
10. Alnylam Pharmaceuticals, Inc;
11. Amberstone Bioscience;
12. Amgen;
13. aMoon
14. Apotex;
15. Arcadia Biosciences, Inc;
16. Arsenal Medical;
17. Artificial Cell Technology, Inc;
18. Avalon-Globocare;
19. Bai Biosciences;
20. BASF Corporation;
21. Bayer;
22. Balzan Foundation;
23. Bexson Biomedical
24. Bilayer Therapeutics;
25. Biogen;
26. BioInnovation Institute (Novo Nordisk Founden);
27. BioTE Medical;
28. Blackrock;
29. Blackstone (Formerly Clarus);
30. Boston Children's Hospital;
31. CBC Group Investment Mgmt Group;
32. Celanese;
33. Celero;
34. Cellink/BICO
35. Cellomics Technology, Llc;
36. Cellular Biomedical;
37. CE&N/ ACS
38. Charles River Laboratories, Inc.;
39. Clontech Laboratories;
40. Combined Therapeutics ("CTx");
41. Conference Forum;
42. Cornell University;
43. Crispr Therapeutics Ag;
44. Crown Bioscience Inc.;
45. Daré Biosciences (Formerly Microchips Biotech, Juniper Pharmaceuticals, and Columbia Laboratories);
46. Daros, Inc.
47. DeepBiome;
48. Dewpoint Therapeutics;
49. Dispendix;
50. Eagle Pharmaceuticals;
51. Earli;
52. Edigene Biotechnology, Inc.;
53. Editas Medicine, Inc.;
54. ELC (Estee Lauder Companies);
55. Eli Lilly;
56. Eisai Inc.;
57. Entrega;
58. EpiBone
59. Establishment Labs, SA.
60. Everlywell;
61. Evox Therapeutics, Ltd.;
62. Fate
63. Flagship Pioneering;
64. Frequency Therapeutics, Inc.;
65. GeneLeap Biotech
66. Genemedicine Co Lmtd;
67. GenScript USA Inc;

2023 Competing Interests Disclosure

From FY 2018 to the present, Dr. Robert Langer receives licensing fees (to patents in which he was an inventor on) from, invested in, consults (or was on Scientific Advisory Boards or Boards of Directors) for, lectured (and received a fee), or conducts sponsored research at MIT for which he was not paid for the following entities:

68. Geneo Medicine;
69. GENUV;
70. Glaxosmithkline Llc;
71. Glycobia;
72. Glympse Bio;
73. Goldman Sachs
74. Greenlight Biosciences;
75. HCR (HealthCare Royalty Partners);
76. HKF DNA Technologies;
77. Hopewell Therapeutics;
78. Horizon Discovery Group Plc;
79. Humacyte, Inc.;
80. IBEX Pharmaceuticals, Inc.;
81. Immunai;
82. ImmuneXcite Inc.;
83. Institute of Immunology Co. Ltd;
84. Integrated DNA Technologies, Inc.;
85. InVivo Therapeutics;
86. IxBio;
87. J.R. Simplot Company;
88. Jnana Therapeutics;
89. Kala Pharmaceuticals;
90. Kallyope, Inc.;
91. Kendall Capital;
92. Kensa;
93. Kodikaz Therapeutics;
94. KAST (Korean Academy of Science and Technology);
95. Ksq Therapeutics, Inc.;
96. Kunlun Capital;
97. Landsdowne Labs;
98. LikeMinds;
99. Lonza;
100. Luminopia, Inc.;
101. Luye (Shandong luye);
102. Lyndra Therapeutics;
103. Lyra Therapeutics (Formerly "480 Biomedical");
104. Maurice Marie Janot Award 2020
105. McGovern Institute;
106. Medikinetics Co., Ltd.;
107. Merck;
108. MGH Ragon Institute;
109. Micelle;
110. Moderna Therapeutics;
111. Momenta;
112. Muse Biotechnologies Inc.
113. Mylan;
114. N2Tech;
115. Nanobiosym;
116. Nanobiotix;
117. Neochromosone;
118. Neoteny 4 LLP;
119. NextRNA;
120. Newbridge Ventures LLC;
121. Noveome Biotherapeutics, Inc.;
122. Novo Nordisk;
123. Ohio State University;
124. Olivo (acquired by Shiseido)
125. Ovid Therapeutics;
126. Particles for Humanity;
127. Pfizer, Inc.;
128. Pioneer Hi-Bred International, Inc.;
129. Placon Therapeutics
130. Polaris Partners;
131. Pontifical Academy of Sciences;
132. Portal Instruments;
133. Preceres, Llc (Acquired by Monsanto);
134. PrognomIQ Inc.;
135. Pulmatrix;

2023 Competing Interests Disclosure

From FY 2018 to the present, Dr. Robert Langer receives licensing fees (to patents in which he was an inventor on) from, invested in, consults (or was on Scientific Advisory Boards or Boards of Directors) for, lectured (and received a fee), or conducts sponsored research at MIT for which he was not paid for the following entities:

136. PureTech;
137. Quris
138. ReLive;
139. Rensselaer Polytechnic Institute/ Department of Chemical and Biological Engineering
140. Reprocell Usa, Inc. (Formerly Stemgent);
141. Replay Bio;
142. Rubius Therapeutics;
143. Satellite Bio;
144. SBEF (Seoul Bio Economy Forum);
145. Secant Medical, Inc.;
146. Seer, Inc.;
147. Selecta Biosciences;
148. Senses LLC;
149. Setsuro Tech Inc.;
150. Seventh Sense Biosystems, Inc.;
151. Shenzhen Rice Life Technology, Ltd;
152. Shire Ag;
153. Sigilon;
154. Sigma Aldrich Co. Llc;
155. Sio2;
156. Ske S.R.L.;
157. Soil Culture Solutions Llc (Dba Soilcea);
158. Souffle Therapeutics
159. SQZ Biotechnologies;
160. StemBioSys, Inc.;
161. SuonoBio;
162. T2 Biosystems;
163. Taconic Biosciences, Inc. (formerly Taconic Farms);
164. Taiwania Capital (Bio-Asia Taiwan Symposium);
165. TARA;
166. Tarveda Therapeutics;
167. Teal Bio
168. Terasaki Institute
169. Tesio Pharmaceuticals
170. Third Rock Ventures;
171. Tiba Biotech LLC;
172. TissiuM (formerly"Gecko");
173. Transgenic Inc.;
174. Translate Bio (Formerly Rana Therapeutics, Inc.);
175. Trilink Biotechnologies, Inc.;
176. Unilever (Living Proof);
177. University of Bergen, Norway (Falch Lecture Honorarium);
178. VasoRX;
179. Verseau Therapeutics, Inc.;
180. Virex Health
181. Vitakey;
182. Vivtex Corporation;
183. Westlake University;
184. Whitehead Institute;
185. Wiki Foods;
186. Xenter;
187. YourBio (Formerly 7th Sense Biosystems)
188. Yz Biosciences (Guangzhou) Inc.;
189. Zenomics;
190. ZWI Therapeutics

REFERENCES

1. S. Correa, A. K. Grosskopf, H. Lopez Hernandez, D. Chan, A. C. Yu, L. M. Stapleton, E. A. Appel, Translational applications of hydrogels. *Chem. Rev.* **121**, 11385–11457 (2021).

2. H. Yuk, J. Wu, X. Zhao, Hydrogel interfaces for merging humans and machines. *Nat. Rev. Mater.* **7**, 935–952 (2022).

3. K. Y. Lee, D. J. Mooney, Hydrogels for tissue engineering. *Chem. Rev.* **101**, 1869–1880 (2001).

4. Y. S. Zhang, A. Khademhosseini, Advances in engineering hydrogels. *Science* **356**, eaaf3627 (2017).

5. J. Li, A. Celiz, J. Yang, Q. Yang, I. Wamala, W. Whyte, B. Seo, N. Vasilyev, J. Vlassak, Z. Suo, D. J. Mooney, Tough adhesives for diverse wet surfaces. *Science* **357**, 378–381 (2017).

6. H. Yuk, C. E. Varela, C. S. Nabzdyk, X. Mao, R. F. Padera, E. T. Roche, X. Zhao, Dry double-sided tape for adhesion of wet tissues and devices. *Nature* **575**, 169–174 (2019).

7. B. R. Freedman, A. Kuttler, N. Beckmann, S. Nam, D. Kent, M. Schuleit, F. Ramazani, N. Accart, A. Rock, J. Li, M. Kurz, A. Fisch, T. Ullrich, M. W. Hast, Y. Tinguely, E. Weber, D. J. Mooney, Enhanced tendon healing by a tough hydrogel with an adhesive side and high drug-loading capacity. *Nat. Biomed. Eng.* **6**, 1167–1179 (2022).

8. H. Yuk, B. Lu, X. Zhao, Hydrogel bioelectronics. *Chem. Soc. Rev.* **48**, 1642–1667 (2019).

9. K. Sagdic, E. Fernández-Lavado, M. Mariello, O. Akouissi, S. P. Lacour, Hydrogels and conductive hydrogels for implantable bioelectronics. *MRS Bull.* **48**, 495–505 (2023).

10. T. Zhou, H. Yuk, F. Hu, J. Wu, F. Tian, H. Roh, Z. Shen, G. Gu, J. Xu, B. Lu, X. Zhao, 3D printable high-performance conducting polymer hydrogel for all-hydrogel bioelectronic interfaces. *Nat. Mater.* **22**, 895–902 (2023).

11. S. Jin, H. Choi, D. Seong, C.-L. You, J.-S. Kang, S. Rho, W. B. Lee, D. Son, M. Shin, Injectable tissue prosthesis for instantaneous closed-loop rehabilitation. *Nature* **623**, 58–65 (2023).

12. B. V. Slaughter, S. S. Khurshid, O. Z. Fisher, A. Khademhosseini, N. A. Peppas, Hydrogels in regenerative medicine. *Adv. Mater.* **21**, 3307–3329 (2009).

13. S. Nam, B. R. Seo, A. J. Najibi, S. L. McNamara, D. J. Mooney, Active tissue adhesive activates mechanosensors and prevents muscle atrophy. *Nat. Mater.* **22**, 249–259 (2023).

14. J. Li, D. J. Mooney, Designing hydrogels for controlled drug delivery. *Nat. Rev. Mater.* **1**, 1–17 (2016).

15. N. A. Peppas, Hydrogels and drug delivery. *Curr. Opin. Colloid Interface Sci.* **2**, 531–537 (1997).

16. J. N. Chu, G. Traverso, Foundations of gastrointestinal-based drug delivery and future developments. *Nat. Rev. Gastroenterol. Hepatol.* **19**, 219–238 (2022).

17. M. A. Bochenek, O. Veiseh, A. J. Vegas, J. J. Mc Garrigle, M. Qi, E. Marchese, M. Omami, J. C. Doloff, J. Mendoza-Elias, M. Nourmohammadzadeh, A. Khan, C.-C. Yeh, Y. Xing, D. Isa, S. Ghani, J. Li, C. Landry, A. R. Bader, K. Olejnik, M. Chen, J. Hollister-Lock, Y. Wang, D. L. Greiner, G. C. Weir, B. L. Strand, A. M. A. Rokstad, I. Lacik, R. Langer, D. G. Anderson, J. Oberholzer, Alginate encapsulation as long-term immune protection of allogeneic pancreatic islet cells transplanted into the omental bursa of macaques. *Nat. Biomed. Eng.* **2**, 810–821 (2018).

18. B. Ying, K. Nan, Q. Zhu, T. Khuu, H. Ro, S. Qin, S. Wang, K. Jiang, Y. Chen, G. Bao, J. Jenkins, A. Pettinari, J. Kuosmanen, K. Ishida, N. Fabian, A. Lopes, F. Codreanu, J. Morimoto, J. Li, A. Hayward, R. Langer, G. Traverso, An electroadhesive hydrogel interface prolongs porcine gastrointestinal mucosal theranostics. *Sci. Transl. Med.* **17**, eadq1975 (2025).

19. S. Nam, D. Mooney, Polymeric tissue adhesives. *Chem. Rev.* **121**, 11336–11384 (2021).

20. S. J. Wu, X. Zhao, Bioadhesive technology platforms. *Chem. Rev.* **123**, 14084–14118 (2023).

21. L. Zhang, Z. Cao, T. Bai, L. Carr, J.-R. Ella-Menye, C. Irvin, B. D. Ratner, S. Jiang, Zwitterionic hydrogels implanted in mice resist the foreign-body reaction. *Nat. Biotechnol.* **31**, 553–556 (2013).

22. A. J. Vegas, O. Veiseh, J. C. Doloff, M. Ma, H. H. Tam, K. Bratlie, J. Li, A. R. Bader, E. Langan, K. Olejnik, P. Fenton, J. W. Kang, J. Hollister-Locke, M. A. Bochenek, A. Chiu, S. Siebert, K. Tang, S. Jhunjhunwala, S. Aresta-Dasilva, N. Dholakia, R. Thakrar, T. Vietti, M. Chen, J. Cohen, K. Siniakowicz, M. Qi, J. M. Garrigle, A. C. Graham, S. Lyle, D. M. Harlan, D. L. Greiner, J. Oberholzer, G. C. Weir, R. Langer, D. G. Anderson, Combinatorial hydrogel library enables identification of materials that mitigate the foreign body response in primates. *Nat. Biotechnol.* **34**, 345–352 (2016).

23. F. Duck, *Physical Properties Of Tissues: A Comprehensive Reference Book* (Academic Press, 1990).

24. C. F. Guimarães, L. Gasperini, A. P. Marques, R. L. Reis, The stiffness of living tissues and its implications for tissue engineering. *Nat. Rev. Mater.* **5**, 351–370 (2020).

25. N. Li, Y. Li, Z. Cheng, Y. Liu, Y. Dai, S. Kang, S. Li, N. Shan, S. Wai, A. Ziaja, Y. Wang, J. Strzalka, W. Liu, C. Zhang, X. Gu, J. A. Hubbell, B. Tian, S. Wang, Bioadhesive polymer semiconductors and transistors for intimate biointerfaces. *Science* **381**, 686–693 (2023).

26. S. Tarafder, G. Y. Park, J. Felix, C. H. Lee, Bioadhesives for musculoskeletal tissue regeneration. *Acta Biomater.* **117**, 77–92 (2020).

27. Y. Wang, K. Jia, C. Xiang, J. Yang, X. Yao, Z. Suo, Instant, tough, noncovalent adhesion. *ACS Appl. Mater. Interfaces* **11**, 40749–40757 (2019).

28. M. H. Ho, Q. van Hilst, X. Cui, Y. Ramaswamy, T. Woodfield, J. Rnjak-Kovacina, S. G. Wise, K. S. Lim, From adhesion to detachment: Strategies to design tissue-adhesive hydrogels. *Adv. NanoBiomed. Res.* **4**, 2300090 (2024).

29. Y. Dai, S. Wai, P. Li, N. Shan, Z. Cao, Y. Li, Y. Wang, Y. Liu, W. Liu, K. Tang, Y. Liu, M. Hua, S. Li, N. Li, S. Chatterji, H. C. Fry, S. Lee, C. Zhang, M. Weires, S. Sutyak, J. Shi, C. Zhu, J. Xu, X. Gu, B. Tian, S. Wang, Soft hydrogel semiconductors with augmented biointeractive functions. *Science* **386**, 431–439 (2024).

30. L. E. Jansen, L. D. Amer, E. Y. T. Chen, T. V. Nguyen, L. S. Saleh, T. Emrick, W. F. Liu, S. J. Bryant, S. R. Peyton, Zwitterionic PEG-PC hydrogels modulate the foreign body response in a modulus-dependent manner. *Biomacromolecules* **19**, 2880–2888 (2018).

31. B. R. Freedman, J. A. Cintron Cruz, P. Kwon, M. Lee, H. M. Jeffers, D. Kent, K. C. Wu, J. C. Weaver, D. J. Mooney, Instant tough adhesion of polymer networks. *Proc. Natl. Acad. Sci. U.S.A.* **121**, e2304643121 (2024).

32. J. Deng, H. Yuk, J. Wu, C. E. Varela, X. Chen, E. T. Roche, C. F. Guo, X. Zhao, Electrical bioadhesive interface for bioelectronics. *Nat. Mater.* **20**, 229–236 (2021).

33. W. Zhang, R. Wang, Z. M. Sun, X. Zhu, Q. Zhao, T. Zhang, A. Cholewinski, F. K. Yang, B. Zhao, R. Pinnaratip, P. K. Forooshani, B. P. Lee, Catechol-functionalized hydrogels: Biomimetic design, adhesion mechanism, and biomedical applications. *Chem. Soc. Rev.* **49**, 433–464 (2020).

34. B. Xue, J. Gu, L. Li, W. Yu, S. Yin, M. Qin, Q. Jiang, W. Wang, Y. Cao, Hydrogel tapes for fault-tolerant strong wet adhesion. *Nat. Commun.* **12**, 7156 (2021).

35. N. Li, S. Kang, Z. Liu, S. Wai, Z. Cheng, Y. Dai, A. Solanki, S. Li, Y. Li, J. Strzalka, M. J. V. White, Y.-H. Kim, B. Tian, J. A. Hubbell, S. Wang, Immune-compatible designs of semiconducting polymers for bioelectronics with suppressed foreign-body response. *Nat. Mater.* **25**, 124–132 (2026).

36. S. Mukherjee, B. Kim, L. Y. Cheng, M. D. Doerfert, J. Li, A. Hernandez, L. Liang, M. I. Jarvis, P. D. Rios, S. Ghani, I. Joshi, D. Isa, T. Ray, T. Terlier, C. Fell, P. Song, R. N. Miranda, J. Oberholzer, D. Y. Zhang, O. Veiseh, Screening hydrogels for antifibrotic properties by implanting cellularly barcoded alginates in mice and a non-human primate. *Nat. Biomed. Eng.* **7**, 867–886 (2023).

37. D. Dong, C. Tsao, H.-C. Hung, F. Yao, C. Tang, L. Niu, J. Ma, J. M. Arthur, A. Sinclair, K. Wu, P. Jain, M. R. Hansen, D. Ly, S. G.-H. Tang, T. M. Luu, P. Jain, S. Jiang, High-strength and fibrous capsule–resistant zwitterionic elastomers. *Sci. Adv.* **7**, eabc5442 (2021).

38. Q. Liu, A. Chiu, L. Wang, D. An, W. Li, E. Y. Chen, Y. Zhang, Y. Pardo, S. P. McDonough, L. Liu, W. F. Liu, J. Chen, M. Ma, Developing mechanically robust, triazole-zwitterionic hydrogels to mitigate foreign body response (FBR) for islet encapsulation. *Biomaterials* **230**, 119640 (2020).

39. X. Zhao, X. Chen, H. Yuk, S. Lin, X. Liu, G. Parada, Soft materials by design: Unconventional polymer networks give extreme properties. *Chem. Rev.* **121**, 4309–4372 (2021).

40. B. Wu, E. Feng, Y. Liao, H. Liu, R. Tang, Y. Tan, Brush-modified hydrogels: Preparations, properties, and applications. *Chem. Mater.* **34**, 6210–6231 (2022).

41. P. L. Wash, E. Maverick, J. Chiefari, D. A. Lightner, Acid–amide intermolecular hydrogen bonding. *J. Am. Chem. Soc.* **119**, 3802–3806 (1997).

42. J. Macron, B. Bresson, Y. Tran, D. Hourdet, C. Creton, Equilibrium and out-of-equilibrium adherence of hydrogels against polymer brushes. *Macromolecules* **51**, 7556–7566 (2018).

43. H. H. Yu, Z. Suo, A model of wafer bonding by elastic accommodation. *J. Mech. Phys. Solids* **46**, 829–844 (1998).

44. G. J. Lake, A. G. Thomas, The strength of highly elastic materials. *Proc. R. Soc. London Ser. A Math. Phys. Sci.* **300**, 108–119 (1967).

45. P. J. M. Bouten, M. Zonjee, J. Bender, S. T. K. Yauw, H. van Goor, J. C. M. van Hest, R. Hoogenboom, The chemistry of tissue adhesive materials. *Prog. Polym. Sci.* **39**, 1375–1405 (2014).

46. J. Yang, R. Bai, B. Chen, Z. Suo, Hydrogel adhesion: A supramolecular synergy of chemistry, topology, and mechanics. *Adv. Funct. Mater.* **30**, 1901693 (2020).

47. J. Yang, R. Bai, Z. Suo, Topological adhesion of wet materials. *Adv. Mater.* **30**, e1800671 (2018).

48. Y. Gao, J. Chen, X. Han, Y. Pan, P. Wang, T. Wang, T. Lu, A universal strategy for tough adhesion of wet soft material. *Adv. Funct. Mater.* **30**, 2003207 (2020).

49. X. Chen, J. Zhang, G. Chen, Y. Xue, J. Zhang, X. Liang, I. M. Lei, J. Lin, B. B. Xu, J. Liu, Hydrogel bioadhesives with extreme acid-tolerance for gastric perforation repairing. *Adv. Funct. Mater.* **32**, 2202285 (2022).

50. S. Bose, L. R. Volpatti, D. Thiono, V. Yesilyurt, C. M. Gladrigan, Y. Tang, A. Facklam, A. Wang, S. Jhunjhunwala, O. Veiseh, J. Hollister-Lock, C. Bhattacharya, G. C. Weir, D. L. Greiner, R. Langer, D. G. Anderson, A retrievable implant for the long-term encapsulation and survival of therapeutic xenogeneic cells. *Nat. Biomed. Eng.* **4**, 814–826 (2020).

51. J. C. Doloff, O. Veiseh, R. de Mezerville, M. Sforza, T. A. Perry, J. Haupt, M. Jamiel, C. Chambers, A. Nash, S. Aghlara-Fotovat, J. L. Stelzel, S. J. Bauer, S. Y. Neshat, J. Hancock, N. A. Romero, Y. E. Hidalgo, I. M. Leiva, A. M. Munhoz, A. Bayat, B. M. Kinney, H. C. Hodges, R. N. Miranda, M. W. Clemens, R. Langer, The surface topography of silicone breast implants mediates the foreign body response in mice, rabbits and humans. *Nat. Biomed. Eng.* **5**, 1115–1130 (2021).

52. S. Capuani, G. Malgir, C. Y. X. Chua, A. Grattoni, Advanced strategies to thwart foreign body response to implantable devices. *Bioeng. Transl. Med.* **7**, e10300 (2022).

53. M. C. Darnell, J.-Y. Sun, M. Mehta, C. Johnson, P. R. Arany, Z. Suo, D. J. Mooney, Performance and biocompatibility of extremely tough alginate/polyacrylamide hydrogels. *Biomaterials* **34**, 8042–8048 (2013).

54. Y. Tanabe, K. Yasuda, C. Azuma, H. Taniguro, S. Onodera, A. Suzuki, Y. M. Chen, J. P. Gong, Y. Osada, Biological responses of novel high-toughness double network hydrogels in muscle and the subcutaneous tissues. *J. Mater. Sci. Mater. Med.* **19**, 1379–1387 (2008).

55. D. Zhang, Q. Chen, Y. Bi, H. Zhang, M. Chen, J. Wan, C. Shi, W. Zhang, J. Zhang, Z. Qiao, J. Li, S. Chen, R. Liu, Bio-inspired poly-DL-serine materials resist the foreign-body response. *Nat. Commun.* **12**, 5327 (2021).

56. W. J. Jeang, M. A. Bochenek, S. Bose, Y. Zhao, B. M. Wong, J. Yang, A. L. Jiang, R. Langer, D. G. Anderson, Silicone cryogel skeletons enhance the survival and mechanical integrity of hydrogel-encapsulated cell therapies. *Sci. Adv.* **10**, eadk5949 (2024).

57. S. Ponce, G. Orive, R. Hernández, A. R. Gascón, J. L. Pedraz, B. J. de Haan, M. M. Faas, H. J. Mathieu, P. de Vos, Chemistry and the biological response against immunoisolating alginate–polycation capsules of different composition. *Biomaterials* **27**, 4831–4839 (2006).

58. J.-Y. Sun, X. Zhao, W. R. Illeperuma, O. Chaudhuri, K. H. Oh, D. J. Mooney, J. J. Vlassak, Z. Suo, Highly stretchable and tough hydrogels. *Nature* **489**, 133–136 (2012).

59. J. Li, Z. Suo, J. J. Vlassak, Stiff, strong, and tough hydrogels with good chemical stability. *J. Mater. Chem. B* **2**, 6708–6713 (2014).

60. N. Noskovicova, R. Schuster, S. van Putten, M. Ezzo, A. Koehler, S. Boo, N. M. Coelho, D. Griggs, P. Ruminski, C. A. McCulloch, B. Hinz, Suppression of the fibrotic encapsulation of silicone implants by inhibiting the mechanical activation of pro-fibrotic TGF-β. *Nat. Biomed. Eng.* **5**, 1437–1456 (2021).

61. O. Veiseh, J. C. Doloff, M. Ma, A. J. Vegas, H. H. Tam, A. R. Bader, J. Li, E. Langan, J. Wyckoff, W. S. Loo, S. Jhunjhunwala, A. Chiu, S. Siebert, K. Tang, J. Hollister-Lock, S. Aresta-Dasilva, M. Bochenek, J. Mendoza-Elias, Y. Wang, M. Qi, D. M. Lavin, M. Chen, N. Dholakia, R. Thakrar, I. Lacík, G. C. Weir, J. Oberholzer, D. L. Greiner, R. Langer, D. G. Anderson, Size-and shape-dependent foreign body immune response to materials implanted in rodents and non-human primates. *Nat. Mater.* **14**, 643–651 (2015).

62. W. J. Jeang, B. M. Wong, Y. Zhao, R. S. Manan, A. L. Jiang, S. Bose, E. Collins, P. M. Mullen, J.-G. Rosenboom, S. Lathwal, R. Langer, D. G. Anderson, Antifouling immunomodulatory copolymer architectures that inhibit the fibrosis of implants. *Adv. Mater.* **37**, e2414743 (2025).

63. G. M. Taboada, K. Yang, M. J. N. Pereira, S. S. Liu, Y. Hu, J. M. Karp, N. Artzi, Y. Lee, Overcoming the translational barriers of tissue adhesives. *Nat. Rev. Mater.* **5**, 310–329 (2020).

64. T. R. Kyriakides, H.-J. Kim, C. Zheng, L. Harkins, W. Tao, E. Deschenes, Foreign body response to synthetic polymer biomaterials and the role of adaptive immunity. *Biomed. Mater.* **17**, 022007 (2022).

65. S. P. Lacour, G. Courtine, J. Guck, Materials and technologies for soft implantable neuroprostheses. *Nat. Rev. Mater.* **1**, 1–14 (2016).

66. O. Chaudhuri, J. Cooper-White, P. A. Janmey, D. J. Mooney, V. B. Shenoy, Effects of extracellular matrix viscoelasticity on cellular behaviour. *Nature* **584**, 535–546 (2020).

67. E. E. Charrier, K. Pogoda, R. G. Wells, P. A. Janmey, Control of cell morphology and differentiation by substrates with independently tunable elasticity and viscous dissipation. *Nat. Commun.* **9**, 449 (2018).

68. S. Wu, M. Hua, Y. Alsaid, Y. Du, Y. Ma, Y. Zhao, C.-Y. Lo, C. Wang, D. Wu, B. Yao, J. Strzalka, H. Zhou, X. Zhu, X. He, Poly (vinyl alcohol) hydrogels with broad-range tunable mechanical properties via the Hofmeister effect. *Adv. Mater.* **33**, e2007829 (2021).

69. J. Kim, G. Zhang, M. Shi, Z. Suo, Fracture, fatigue, and friction of polymers in which entanglements greatly outnumber cross-links. *Science* **374**, 212–216 (2021).

70. F. Lorandi, M. Fantin, Y. Wang, A. A. Isse, A. Gennaro, K. Matyjaszewski, Atom transfer radical polymerization of acrylic and methacrylic acids: Preparation of acidic polymers with various architectures. *ACS Macro Lett.* **9**, 693–699 (2020).

71. R. S. Rivlin, A. G. Thomas, Rupture of rubber. I. Characteristic energy for tearing. *J. Polym. Sci.* **10**, 291–318 (1953).

72. Y. Wang, X. Yang, G. Nian, Z. Suo, Strength and toughness of adhesion of soft materials measured in lap shear. *J. Mech. Phys. Solids* **143**, 103988 (2020).

73. A. N. Azadani, P. B. Matthews, L. Ge, Y. Shen, C.-S. Jhun, T. S. Guy, E. E. Tseng, Mechanical properties of surgical glues used in aortic root replacement. *Ann. Thorac. Surg.* **87**, 1154–1160 (2009).

74. J. O. Zoppe, N. C. Ataman, P. Mocny, J. Wang, J. Moraes, H.-A. Klok, Surface-initiated controlled radical polymerization: State-of-the-art, opportunities, and challenges in surface and interface engineering with polymer brushes. *Chem. Rev.* **117**, 1105–1318 (2017).

75. J. Tang, J. Li, J. J. Vlassak, Z. Suo, Fatigue fracture of hydrogels. *Extreme Mech. Lett.* **10**, 24–31 (2017).

76. P. G. De Gennes, Scaling theory of polymer adsorption. *J. Phys.* **37**, 1445–1452 (1976).

77. S. Alexander, Adsorption of chain molecules with a polar head a scaling description. *J. Phys.* **38**, 983–987 (1977).

78. W.-L. Chen, R. Cordero, H. Tran, C. K. Ober, *50th anniversary perspective*: Polymer brushes: Novel surfaces for future materials. *Macromolecules* **50**, 4089–4113 (2017).

79. P. J. Flory, *Principles of Polymer Chemistry* (Cornell Univ. Press, 1953).
